# CD45 limits early Natural Killer cell development

**DOI:** 10.1111/imcb.12701

**Published:** 2023-10-19

**Authors:** Lizeth G Meza Guzman, Craig D Hyland, Grace M Bidgood, Evelyn Leong, Zihan Shen, Wilford Goh, Jai Rautela, James E Vince, Sandra E Nicholson, Nicholas D Huntington

**Affiliations:** ^1^ The Walter and Eliza Hall Institute of Medical Research Parkville VIC Australia; ^2^ Department of Medical Biology The University of Melbourne Melbourne VIC Australia; ^3^ Department of Biochemistry and Molecular Biology, Biomedicine Discovery Institute Monash University Clayton VIC Australia

**Keywords:** CD45, hematopoiesis, lymphocytes, Natural Killer cells, NK progenitor

## Abstract

The clinical development of Natural Killer (NK) cell‐mediated immunotherapy marks a milestone in the development of new cancer therapies and has gained traction due to the intrinsic ability of the NK cell to target and kill tumor cells. To fully harness the tumor killing ability of NK cells, we need to improve NK cell persistence and to overcome suppression of NK cell activation in the tumor microenvironment. The trans‐membrane, protein tyrosine phosphatase CD45, regulates NK cell homeostasis, with the genetic loss of CD45 in mice resulting in increased numbers of mature NK cells. This suggests that CD45‐deficient NK cells might display enhanced persistence following adoptive transfer. However, we demonstrate here that adoptive transfer of CD45‐deficiency did not enhance NK cell persistence in mice, and instead, the homeostatic disturbance of NK cells in CD45‐deficient mice stemmed from a developmental defect in the progenitor population. The enhanced maturation within the CD45‐deficient NK cell compartment was intrinsic to the NK cell lineage, and independent of the developmental defect. CD45 is not a conventional immune checkpoint candidate, as systemic loss is detrimental to T and B cell development, compromising the adaptive immune system. Nonetheless, this study suggests that inhibition of CD45 in progenitor or stem cell populations may improve the yield of *in vitro* generated NK cells for adoptive therapy.

## Graphical abstract

In this study, we provide evidence that the increased NK cell expansion in mice, with a global loss of CD45, stemmed from a defect in an early progenitor/stem cell population, rather than from an intrinsic effect of CD45 on NK cell proliferation or survival. In addition, we demonstrated that the NK cell hyperplasia in *Cd45* ^‐^^/^^‐^ mice was not due to differential responsiveness to IL‐15, a cytokine required for NK cell survival, proliferation and maturation, or due to increased systemic IL‐15.

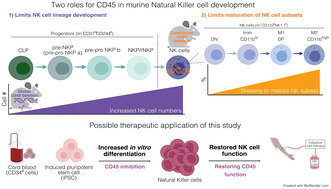

## INTRODUCTION

Leucocyte common antigen CD45 (Ly5; encoded by the *Ptprc* gene) is a prototypic transmembrane receptor‐like protein tyrosine phosphatase (PTP) expressed on all nucleated hematopoietic cells and is required for normal lymphocyte development.^1^, ^2^, ^3^ There are two proposed ligands for CD45, CD22 a B cell restricted transmembrane glycoprotein and galectin‐1 a carbohydrate‐binding protein with an affinity for β‐galactosides.^4^, ^5^, ^6^ To date, galectin‐1 is the only physiological ligand that results in inhibition of PTPase activity upon CD45 engagement.^5^, ^7^ Nonetheless, efforts to discover a ligand for CD45 that positively modulates PTPase activity are still ongoing.

CD45 has the ability to influence multiple signaling pathways through its various biochemical functions, the dominant one being dephosphorylation of the C‐terminal negative regulatory tyrosine in the Src‐family kinases, consequently activating Src kinases upon T and B cell receptor (TCR; BCR) engagement.^8^, ^9^, ^10^, ^11^ It is therefore an indispensable positive regulator of T cell antigen receptor signaling in cell lines, mouse models and humans.^12^, ^13^, ^14^, ^15^ CD45 deficiency in humans results in a form of severe combined immunodeficiency that is characterized by decreased T cell numbers, normal, decreased or increased B cell numbers, and normal or increased numbers of NK cells.^16^, ^17^, ^18^

In addition to its role in TCR and BCR activation,^8^, ^9^, ^10^, ^11^ CD45 has been reported to activate Syk, JNK and p38, downstream of Ly49D activation in NK cells.^19^, ^20^ Lastly, CD45 has been shown to suppress Janus kinase (JAK) activity, resulting in negative regulation of cytokine receptor signaling.^21^

In mice, the *Ptprc* gene consists of 34 exons that encode an extracellular domain (exons 1–12), interdomain (exons 13–14), transmembrane domain (exon 15) and an intracellular cytoplasmic domain (exons 16–34). T, B and NK cells express multiple CD45 isoforms, that are differentially expressed during developmental stages.^3^ CD45 isoform switching occurs as lymphoid cells differentiate into T, B and NK cells, with less mature populations expressing larger isoforms, suggesting a lower activation threshold compared with more mature populations expressing the smaller isoforms.^22^

Three CD45‐deficient models have been generated in mice by independently targeting exons 6,^8^ 9^9^ and 12.^10^ All three models display defects in thymic development mediated by increased apoptosis and dysfunctional pre‐TCR and TCR signaling.^11^ Deletion of exon 6 resulted in complete abrogation of CD45 expression on all B cells, while a small population of thymic T cells (3–5%) retained CD45 expression.^8^ Deletion of exon 9 resulted in the complete loss of CD45 on B and T cells.^9^ Lastly, targeting exon 12, which encodes part of the extracellular domain in all isoforms, resulted in the complete loss of CD45 surface expression on all cell types.^10^ Although this CD45 null mouse strain (*Cd45* ^*−/−*^) lacks membrane bound CD45, immunoblotting revealed the presence of a truncated protein (~150 kDa).^10^ Despite this, the exon 12‐targeted *Cd45* ^*−/−*^ phenotype is identical to the exon 9‐targeted model.

CD45‐deficient mice lack mature T and B cells.^8^, ^9^, ^10^, ^11^ T cell loss is due to dysfunctional TCR signaling leading to a reduction in double positive and single positive thymocytes, as well as a defect in negative selection following antigen stimulation.^23^ Thus, the CD45‐deficient T cells that do develop and reach the periphery are highly autoreactive. B cell development halts at T2 transitional stage in the spleen as B cells fail to express IgD.^24^

Although CD45 is required for T and B cell development, this does not appear to be the case for NK cells.^19^, ^25^ Splenic NK cells in *Cd45* ^*−/−*^ mice were increased 4–5‐fold and actively dividing, compared with NK cells in control mice, suggesting that CD45 is a regulator of NK cell homeostasis, functioning to limit NK cell numbers.^19^, ^26^ Additionally, splenic NK cells from *Cd45* ^*−/−*^ mice retained cytotoxic ability but exhibited defective IFNγ production.^19^, ^27^

The *in vivo* expansion and/or persistence of NK cells in CD45‐deficient mice suggests that CD45 is a potential target for immunotherapies designed to enhance NK cell anti‐tumor activity. Adoptive NK cell therapies are considered safe, with no cytokine release syndrome or graft‐versus‐host disease observed in clinical trials so far and have been successfully used against relapsed or refractory CD19‐positive cancers (non‐Hodgkin's lymphoma or chronic lymphocytic leukemia.^28^, ^29^ Targeting CD45 in NK cell‐based immune cell therapy has the potential to bypass the severe T and B cell immunosuppression expected with whole body inhibition of CD45 activity. Here we investigated the role that CD45 plays in regulating NK cell homeostasis and development.

## RESULTS

Mice lacking CD45 have reduced numbers of mature T and B cells, but increased numbers of mature M2 stage NK cells with enhanced *in vitro* cytotoxic capability.^19^, ^26^ However, previous studies did not address whether the increased NK cell maturation in *Cd45* ^*−/−*^ mice was related to IL‐15 signaling, or at what stage in NK development CD45 limited expansion of the NK cell pool.

**Figure 1:**
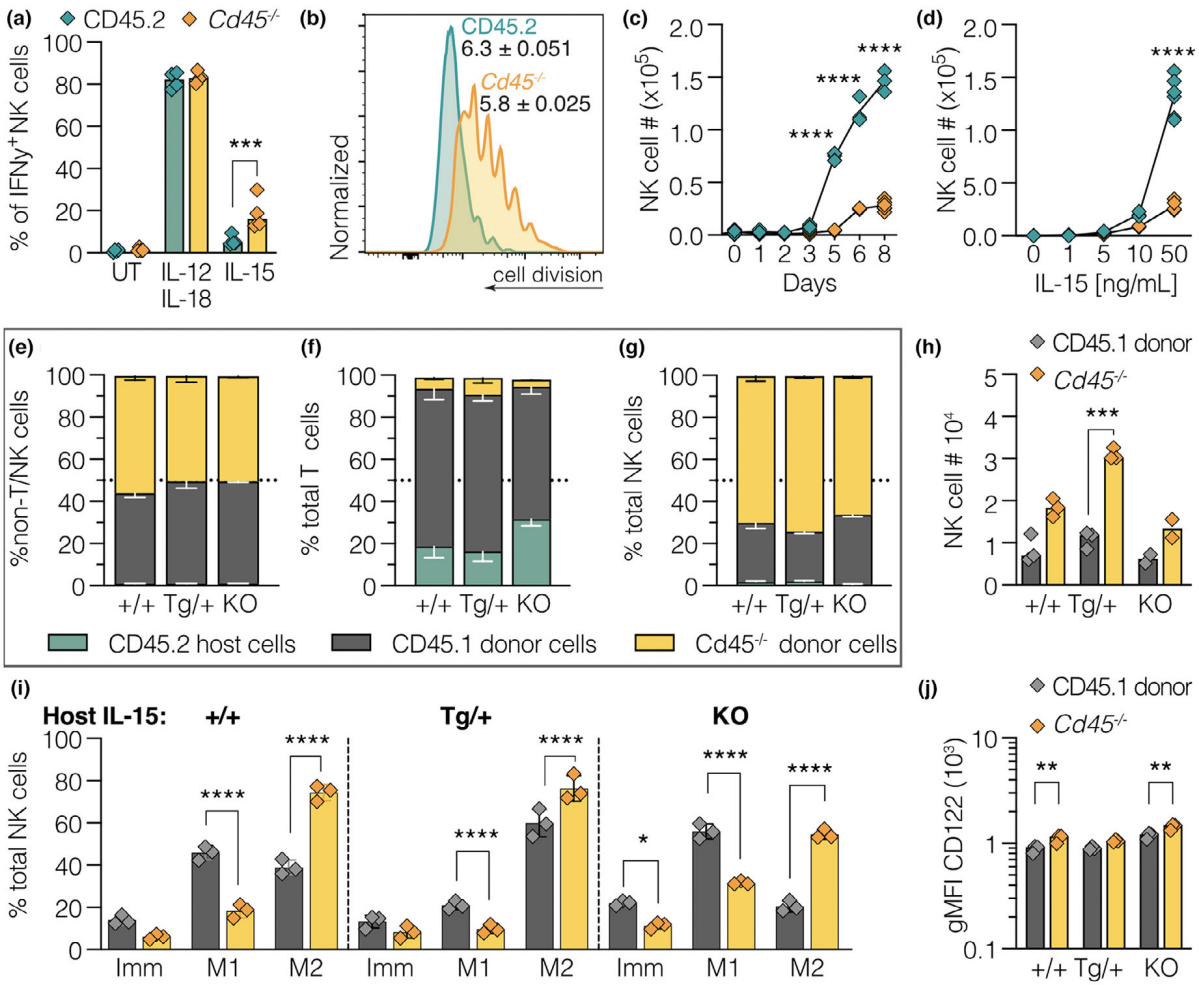
The *in vivo* expansion of *Cd45* ^*−/−*^ NK cells is not dependent on IL‐15. **(a)** Splenocytes from 6–8‐week‐old *Cd45* ^+/+^ (CD45.2; green) or *Cd45* ^*−/−*^ (yellow; littermates) male mice, were untreated (UT) or treated for 4 h with cytokines, and NK cells (CD3^−^TCRß^−^NK1.1^+^NKp46^+^) analyzed for intracellular IFNγ production. Percentage of IFNγ^+^ NK cells. Data are representative of two independent experiments. Each symbol represents cells from an individual mouse. **(b–d)** Splenic NK cells from 6–8‐week‐old *Cd45* ^*+/+*^ (CD45.2; green) or *Cd45* ^*−/−*^ (yellow; littermates) mice were isolated and labeled with CellTraceViolet (CTV; 5 μm), prior to culturing for 8 days with recombinant human IL‐15 (0, 1, 5, 10, 50 ng mL^−1^). Viable cell numbers were analyzed daily. **(b)** Example histograms depicting CTV‐labeled NK cell division peaks following 8‐day culture in 50 ng mL^−1^ IL‐15. **(c)** Absolute numbers of NK cells with 1 to 8 days culture in 50 ng mL^−1^ hIL‐15. **(d)** Absolute numbers of NK cells at day 8 with titration of IL‐15 levels. **(c, d)** Mean ± s.d. combined data from three independent experiments; each symbol represents an individual mouse, *n* = 6 mice (male and female). Note the data corresponding to 8‐day culture in 50 ng mL^−1^ IL‐15 is plotted in both **c** and **d**. **(e–j)** Bone marrow was harvested from 6–8‐week‐old female control C57BL/6 (CD45.1; Ly5.1; gray) and *Cd45* ^*−/−*^ (yellow) mice, mixed in a 1:1 ratio and a single cell suspension transplanted into lethally irradiated IL‐15 Tg^+/+^, IL‐15 Tg^T/+^ and IL‐15^−/−^ CD45.2/Ly5.2 host female mice to generate competitive bone marrow chimeras. 8‐week post‐transplantation, spleens were analyzed for reconstitution by CD45.1 and *Cd45* ^*−/−*^ donor cells: **(e)** % non‐T/NK cells (CD3^−^NK1.1^−^), **(f)** % pan T cells (CD3^+^), **(g)** % NK cells (CD3^−^/NK1.1^+^/Nk46^+^) and **(h)** total NK cell numbers. **(i)** NK maturation profile (Imm: CD11b^−^KLRG1^−^, M1: CD11b^+^ KLRG1^−^, M2: CD11b^+^ KLRG1^+^) and **(j)** surface expression of CD122. Competitive chimeras were generated twice, with at least five hosts per genotype. **(e–g)** *n* = 5 mice. **(h–j)** *n* = 3 mice, **(e–g)** combined data shown. Significance was determined by two‐way ANOVA with Sidak's multiple comparisons test (* < 0.5, ** < 0.01, ** < 0.001, and **** < 0.0001). Representative gating strategy for all flow cytometry experiments in Supplementary figure 2.

IL‐15 is critical for NK cell survival and proliferation and contributes to IFNγ production.^30^, ^31^, ^32^, ^33^, ^34^ Given that CD45 is also known to negatively regulate cytokine receptor signaling,^21^ we explored the interplay between IL‐15 responses and the phenotype of CD45‐deficient mice. Initially, splenic cells from wild‐type C57BL/6 (CD45.2) and *Cd45* ^*−/−*^ mice were stimulated *ex vivo* with IL‐12 and IL‐18, or IL‐15 cytokines. *Cd45* ^*−/−*^ NK cells produced slightly more IFNγ in response to IL‐15 treatment, compared with wild‐type NK cells. No differences were observed with IL‐12 and IL‐18 treatment (Figure 1a and Supplementary figure 2a). Degranulation, as measured by CD107a expression, was not different in IL‐15 treated *Cd45* ^−/−^ NK cells (data not shown).

### *In vitro* proliferation of *Cd45* ^*−/−*^ NK cells is compromised in response to IL‐15

To further assess the role of CD45 downstream of IL‐15 signaling, splenic NK cells were purified from wild‐type and *Cd45* ^*−/−*^ mice, labeled with CTV, and NK cell division tracked over 8 days in the presence of various hIL‐15 concentrations (Figure 1b–d and Supplementary figure 2b). Wild‐type NK cells divided on average 6.3 times (50 ng mL^−1^; 8 days), consistent with previous data.^35^ In contrast, *Cd45* ^*−/−*^ NK cells were not able to reach division 7, and instead divided ~5.8 times, with most cells achieving fewer divisions (Figure 1b). The normalized *Cd45* ^*−/−*^ NK cell cohort number (relative to initial number of cells that undergo division) was also decreased (0.226 ± 0.043 *versus* 0.917 ± 0 0.0367; *Cd45* ^*−/−*^ *versus* wild‐type). At days 5 and 8, the total numbers of *Cd45* ^*−/−*^ NK cells were significantly reduced compared with wild‐type NK cells (~4.6‐fold in 50 ng mL^−1^ IL‐15; Figure 1c, d). This result was not consistent with the described *in vivo* expansion of the NK cell pool in *Cd45* ^*−/−*^ mice.^19^

### CD45 regulation of NK cell homeostasis and maturation is not IL‐15 independent

Although the ability of *Cd45* ^*−/−*^ NK cells to respond to IL‐15 was compromised *ex vivo*, it was not clear whether homeostatic maintenance of NK cells *in vivo* (which also requires IL‐15), would be impacted. To investigate this, bone marrow (BM) cells from *Cd45* ^*−/−*^ (Ly5.1/2^−/−^) and CD45.1 (Ly5.1) mice were mixed (1:1; Supplementary figure 1c) and transplanted into lethally irradiated CD45.2/Ly5.2 wild‐type mice (IL‐15^+/+^; IL‐15tg littermates), IL‐15 transgenic mice (IL‐15tg^T/+^) and IL‐15 deficient mice (IL‐15^−/−^). Note, IL‐15 deficiency is not complete in IL‐15^−/−^ hosts, as transplanted cells can produce IL‐15. Considering the lack of mature T cells and expansion of the NK cell compartment in *Cd45* ^*−/−*^ BM chimeras,^8^, ^9^, ^10^ reconstitution was assessed in the splenic CD3^−^NK1.1^−^ population (non‐T/NK cells), which showed equal reconstitution by *Cd45* ^*−/−*^ and CD45.1 donor cells (Figure 1e and Supplementary figure 1d).

As expected, less than 10% of reconstituted T cells (CD3^+^) were of *Cd45* ^*−/−*^ donor origin (Figure 1f). Lastly, the NK cell compartment in all recipients was dominated by *Cd45* ^*−/−*^ donor cells (> 60%), with a corresponding increase in *Cd45* ^*−/−*^ NK cell number (Figure 1g, h). Both wild‐type and *Cd45* ^*−/−*^ NK donor cell numbers were increased in IL‐15 Tg^T/+^ host mice, but *Cd45* ^*−/−*^ NK donor cells were not selectively enhanced by IL‐15 (1.2‐fold for *Cd45* ^*+/+*^; 1.6‐fold for *Cd45* ^*−/−*^; Figure 1h). The reconstituted *Cd45* ^*−/−*^ NK cells skewed towards an M2 (KLRG1^+^CD11b^+^) maturation profile^26^ (Supplementary figure 2e) in all hosts. This was independent of IL‐15 levels (Figure 1i, Supplementary figure 2e), and consistent with previous reports in chimeric wild‐type mice.^19^ Similarly, *Cd45* ^*−/−*^ cells displayed slightly elevated levels of the IL‐15 receptor beta subunit (IL‐2Rβ; CD122), with no significant difference in CD122 levels in mice bearing an IL‐15 transgene (Figure 1j).

In contrast to the reduced *in vitro* proliferation of *Cd45* ^*−/−*^ NK cells (Figure 1b–d), the enhanced *in vivo* expansion of the *Cd45* ^*−/−*^ NK cell compartment was recapitulated in the mixed bone marrow chimeras. Changes in IL‐15 levels or IL‐15 receptor beta subunit (IL‐2Rβ; CD122) expression had no impact on either the expansion of the *Cd45* ^*−/−*^ NK cell compartment or the skewing towards mature M2 effector NK cells.

### Adoptively transferred *Cd45* ^*−/−*^ NK cells display reduced expansion in lymphocyte depleted mice

Although the competitive bone marrow chimeras confirmed that expansion of the NK cell compartment in *Cd45* ^*−/−*^ mice was intrinsic to hematopoietic cells, the chimeras did not address whether expansion was intrinsic to the NK cell lineage or resulted from a defect at an earlier stage of hematopoiesis. Competitive, adoptive NK cell transfers were performed to determine whether CD45‐deficient NK cells were able to expand and accumulate *in vivo*. *Cd45* ^*−/−*^ and *Cd45* ^+/+^ NK cells were purified, labeled with CTV, mixed (1:1) and adoptively transferred into NK cell‐deficient mice (*Mcl1* ^*fl/fl*^ *Ncr1* ^*iCre/+*^),^36^ T and B cell‐deficient mice (*Rag‐1* ^*−/−*^) or completely alymphoid mice (*Rag‐2* ^*−/−*^ γ *c* ^*−/−*^).

**Figure 2:**
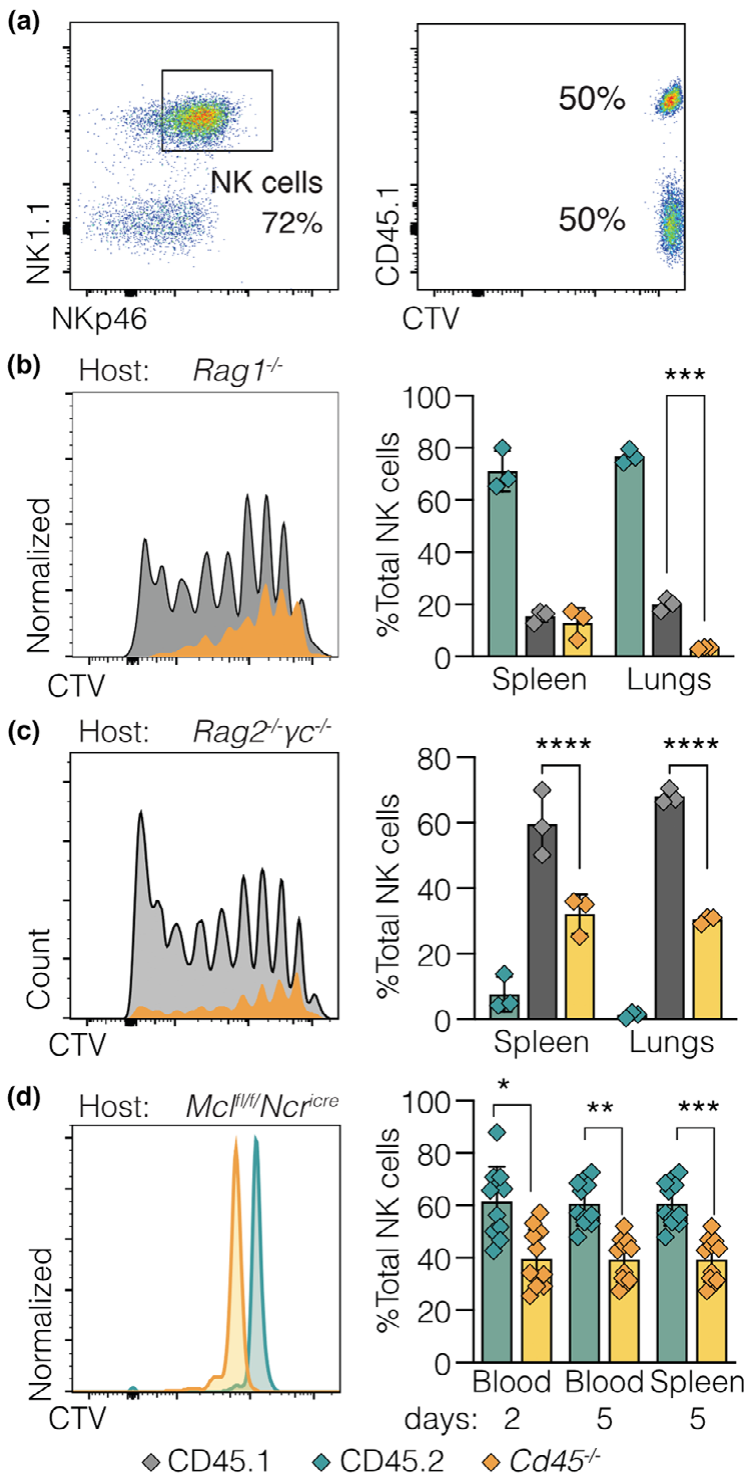
Loss of CD45 compromises the ability of NK cells to expand following adoptive transfer. **(a–c)** Splenic NK cells were enriched from 6–8‐week‐old CD45.1 (Ly5.1; gray) and *Cd45* ^*−/−*^ (Ly5.1/2 null; yellow) female mice by negative depletion, labeled with CTV, and mixed in a 1:1 ratio for I.V. injection into T and B cell‐deficient (*Rag‐1* ^*−/−*^), or completely alymphoid (*Rag‐2* ^*−/−*^γ*c* ^*−/−*^ *)* host female mice (CD45.2; green). **(a)** Confirmation of NK cell enrichment (left panel), and 1:1 ratio of CTV labeled cells prior to injection (right panel). **(b)** *Rag‐1* ^*−/−*^ (*n* = 3 hosts) and **(c)** *Rag‐2* ^*−/−*^γ*c* ^*−/−*^ (*n* = 3 hosts) mice were killed 6 days post‐adoptive transfer, and spleens collected for analysis of NK cell proliferation (left panels) and relative proportions of donor and host cells in the NK cell compartment (right panels). **(d)** Splenic NK cells were enriched from 6–8‐week‐old Cd45.2 (Ly5.2; green) and Cd45^−/−^ (Ly5.1/2 null; yellow) female or male mice by negative depletion, labeled with CTV, and mixed in a 1:1 ratio for I.V. injection into NK cell‐deficient (*Mcl1* ^*fl/fl*^ *Ncr1* ^*Ki/+*^) female or male mice, respectively. Two days post‐adoptive cell transfer, blood samples were analyzed for frequency of transferred NK cells. At day 5 post‐adoptive cell transfer, blood and spleens were collected and analyzed for frequency of transferred NK cells (right panel). Representative histograms are shown on the left. Combined data from three independent experiments; each symbol represents an individual mouse, *n* = 11 (male and female) mice. Statistical significance was determined by two‐way ANOVA with Sidak's multiple comparisons test (* < 0.5, ** < 0.01, *** < 0.001 and **** < 0.0001).

In contrast to the bone marrow chimeras (Figure 1g, h), the expansion of adoptively transferred *Cd45* ^*−/−*^ NK cells was significantly impaired in *Rag‐2* ^*−/−*^ γ *c* ^*−/−*^ and *Rag‐1* ^*−/−*^ hosts, with fewer *Cd45* ^*−/−*^ cells undergoing less cell divisions, than adoptively transferred CD45.1 NK cells (Figure 2a–c). This was consistent with a reduced contribution by transferred *Cd45* ^*−/−*^ cells to the NK cell compartment in spleen and lung, compared with CD45.1/Ly5.1 NK cells (for example, 33% *versus* 66% respectively, in *Rag‐2* ^*−/−*^ γ *c* ^*−/−*^; Figure 2b, c). To further examine the capacity of *Cd45* ^*−/−*^ NK cells to persist *in vivo*, NK cells were adoptively transferred into NK cell‐deficient mice (*Mcl1* ^*fl/fl*^ *Ncr1* ^*iCre/+*^). Again, the relative contribution of *Cd45* ^*−/−*^ cells to the NK cell compartment was significantly less than that observed for wild‐type NK cells (Figure 2d).

These observations were in sharp contrast to the original reported expansion of NK cells,^19^ and the bone marrow chimera data in Figure 1, which suggested that deletion of CD45 conferred an intrinsic proliferative advantage to NK cells. Instead, these data indicated that CD45 acted prior to NK cell development. To test this hypothesis, mice were generated with conditional deletion of *Cd45* in NK cells.

### Conditional deletion of CD45 in NK cells does not disrupt NK cell homeostasis, but does skew maturation towards mature M2 cells

**Figure 3:**
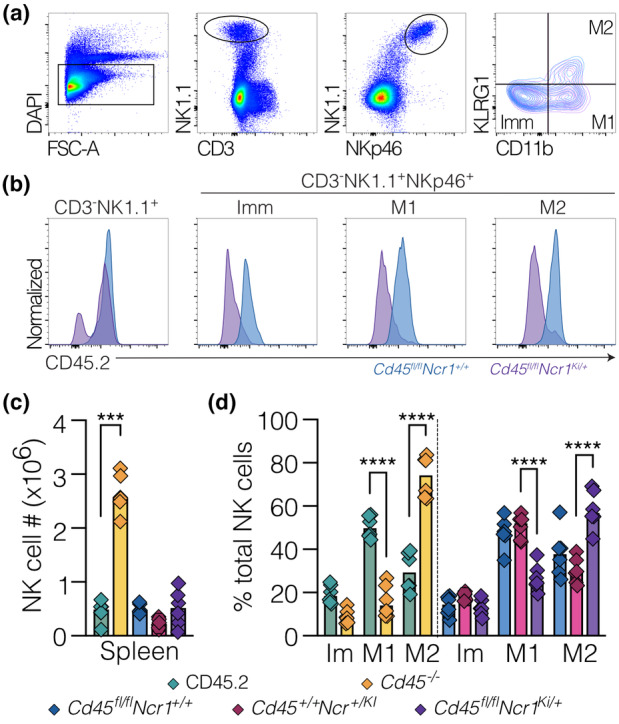
Loss of CD45 in NK cells disrupts NK cell maturation but not homeostasis. **(a)** Flow cytometric analysis confirming conditional deletion of CD45 in NK cells. Bone marrow cells from control mice (*Cd45* ^*+/+*^ *Ncr1* ^*iCre/+*^; blue) and mice with NK deletion of *Cd45* (*Cd45* ^*fl/fl*^ *Ncr1* ^*iCre/+*^; purple) were gated on NK precursor markers (CD3^−^NK1.1^+^), NK cell markers (CD3^−^NK1.1^+^NKp46^+^) followed by NK maturation markers (CD11b and KLRG1) to distinguish immature (Imm) and mature (M1 and M2) NK cells, and analyzed for CD45 expression. **(b)** Histograms are representative of *n* = 8 *Cd45* ^*fl/fl*^ *Ncr1* ^*iCre/+*^ mice and *n* = 7 *Cd45* ^*+/+*^ *Ncr1* ^*iCre/+*^ mice. **(c, d)** Splenic cells from Cd45^+/+^ (CD45.2; green), Cd45^−/−^ (yellow), Cd45^+/+^Ncr1^*iCre*/+^ (blue), Cd45^*fl/fl*^Ncr1^+/+^ (pink), and Cd45^*fl/fl*^Ncr1^*iCre*/+^ (purple) mice were analyzed for NK cell (CD3^−^NK1.1^+^NKp46^+^) numbers and maturation. **(c)** Splenic NK cell numbers. **(d)** Frequencies of splenic NK cell maturation populations based on CD11b and KLRG1 expression. **(c, d)** Combined data from two independent experiments; each symbol represents an individual mouse. *n* = 4–6 (male and female) mice. Statistical significance was determined by two‐way ANOVA with Sidak's multiple comparisons test (*** < 0.001 and **** < 0.0001).

CRISPR/Cas9 gene editing was used to generate mice carrying *Cd45/ptprc* conditional alleles (loxP). Mice carrying the floxed *Cd45* allele (*Cd45* ^*fl/fl*^) were crossed with mice containing the cre recombinase gene under control of the *Ncr1* gene promoter (encoding NKp46; Ncr1^iCre/+^ ^37^), to generate *Cd45* ^*fl/fl*^ *Ncr1* ^*iCre/+*^ mice. This resulted in deletion of C*d45* exon 14 from the immature stage of NK cell development (Supplementary figure 1). Lack of CD45 surface expression on *Cd45* ^*fl/fl*^ *Ncr1* ^*iCre/+*^ NK cells was confirmed by flow cytometry (Figure 3a). Surface expression of CD45 remained unaltered in the lymphocyte (Supplementary figure 3) and myeloid (Supplementary figure 4) compartments. Additionally, T cell (Supplementary figure 5), B cell (Supplementary figure 6) and NK cell (Supplementary figure 7) development was normal in *Cd45* ^*fl/fl*^ *Ncr1* ^*iCre/+*^ mice.

In contrast to global deletion of *Cd45*, conditional deletion of *Cd45* in NK cells did not result in expansion of the NK cell compartment. NK cell numbers in the spleen of *Cd45* ^*fl/fl*^ *Ncr1* ^*iCre/+*^ mice were comparable to *Cd45* ^*fl/fl*^ *Ncr1* ^+/+^ and *Cd45* ^*+/+*^ *Ncr1* ^*iCre/+*^ control mice, and within a normal range (Figure 3c, d). This was not due to an inability to detect NK cells, as a CD45 negative NK population was present in *Cd45* ^*fl/fl*^ *Ncr1* ^*iCre/+*^ mice (CD3^−^NK1.1^+^NKp46^+^) throughout NK cell maturation (Figure 3a, e). This further suggested that the dramatic increase in NK cell numbers observed in *Cd45* ^*−/−*^ mice resulted from changes in early NK development, prior to expression of NKp46.

Interestingly, NK cell maturation does appear to be intrinsically regulated by CD45, as the maturation profile of splenic NK cells from *Cd45* ^*fl/fl*^ *Ncr1* ^*iCre/+*^ mice remained skewed to the more mature M2 population (Figure 3d), consistent with previous observations (Figure 1i).

### CD45 regulates NK cell development

**Figure 4:**
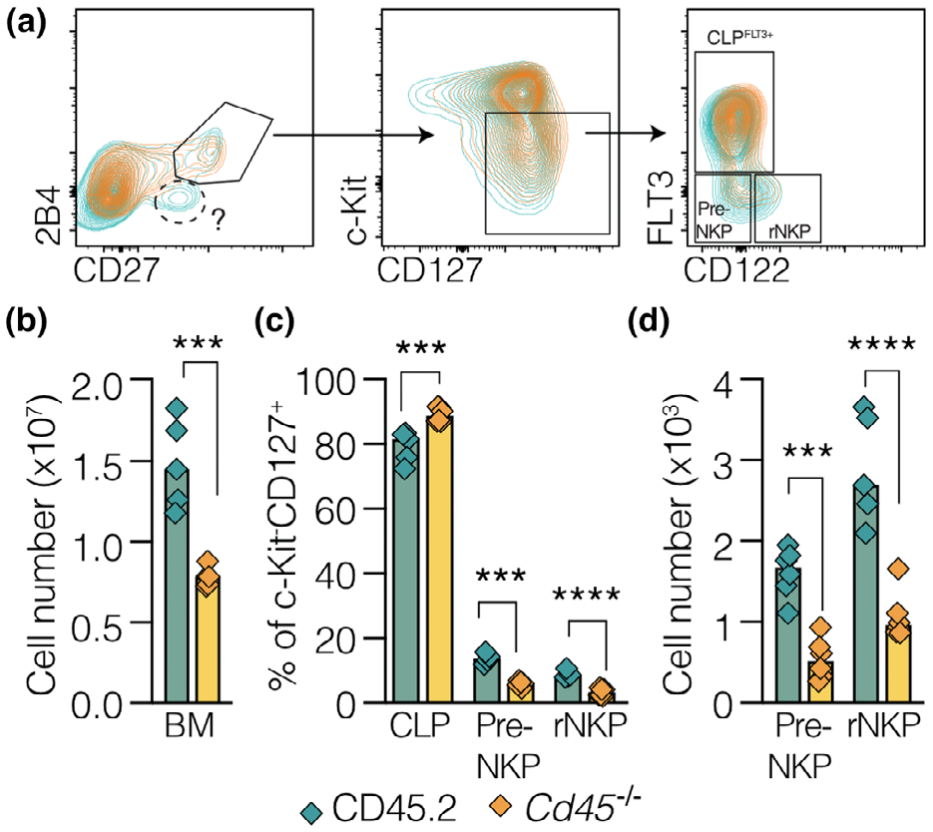
Loss of CD45 reduces NK progenitors in mice. Bone marrow cells were harvested from 6–8‐week‐old male and female *Cd45* ^*+/+*^ (CD45.2; green) and *Cd45* ^*−/−*^ (yellow; littermates) mice and NK cell progenitors analyzed by flow cytometry. **(a)** Gating strategy for analysis of NK progenitors post‐exclusion of other lineages (CD3e, CD8, CD4, CD19, TER119, Ly6G, CD11b, Ly6D and NK1.1). NK cell populations were analyzed based on expression of 2B4, CD27, cKit and CD127. FLT3 expression identifies the common lymphoid progenitor (CLP; CD3e^−^CD8^−^CD4^−^CD19^−^TER119^−^Ly6G^−^CD11b^−^ Ly6D^−^NK1.1^−^; 2B4^+^CD27^+^; cKit^−^CD127^+^; Flt3^+^), while lack of FLT3 expression defines NK progenitors (NKP: CD3e^−^CD8^−^CD4^−^ CD19^−^ TER119^−^Ly6G^−^CD11b^−^Ly6D^−^NK1.1^−^; 2B4^+^CD27^+^; cKit^−^CD127^+^; Flt3^−^). Two NK progenitor populations have been defined, the pre‐NKP (NKP; CD122^−^) and restricted NKP (rNKP: NKP; CD122^+^). An uncharacterized population (Lin^−^2B4^med^CD27^med^) that is missing in Cd45^−/−^ bone marrow is highlighted. **(b)** Enumeration of viable bone marrow (BM) lymphocytes. **(c)** Frequencies of CLP, pre‐NKP and rNKP populations. **(d)** Absolute cell numbers. **(b–d)** Combined data from two independent experiments; each symbol represents an individual mouse (*n* = 3 mice of each gender in each experiment; six in total per genotype). Significance was determined by two‐way ANOVA with Sidak's multiple comparisons test (*** < 0.001 and **** < 0.0001).

To assess early NK development, NK progenitor populations NKP (CD3e^−^CD8^−^CD4^−^CD19^−^TER119^−^Ly6G^−^CD11b^−^Ly6D^−^NK1.1^−^; 2B4^+^CD27^+^; cKit^−^CD127^+^; Flt3^−^), pre‐NKP (NKP; CD122^−^) and rNKP (NKP; CD122^+^) were enumerated in bone marrow of *Cd45* ^*−/−*^ mice (Figure 4a). Notably, the absolute number of viable bone marrow cells in *Cd45* ^*−/−*^ mice was decreased compared with control mice (Figure 4b). In addition, a Lin^−^2B4^med^CD27^med^ population (not previously characterized), was absent in *Cd45* ^*−/−*^ bone marrow (Figure 4a). The common lymphoid progenitor (CLP; Lin^−^2B4^+^CD27^+^ cKit^−^CD127^+^ Flt3^+^) population, which gives rise to the pre‐NKP and rNKP populations, was marginally increased in bone marrow from *Cd45* ^*−/−*^ mice (Figure 4c). Lastly, a ~ 4 and ~ 3‐fold decrease in pre‐NKP and rNKP populations, respectively, was observed in *Cd45* ^*−/−*^ bone marrow (Figure 4c, d). One possibility is that in *Cd45* ^*−/−*^ mice there is a faster transition from the CLP into NK progenitors (pre‐NKP and rNKP), resulting in increased numbers of NK cells (Figure 1i).

**Figure 5:**
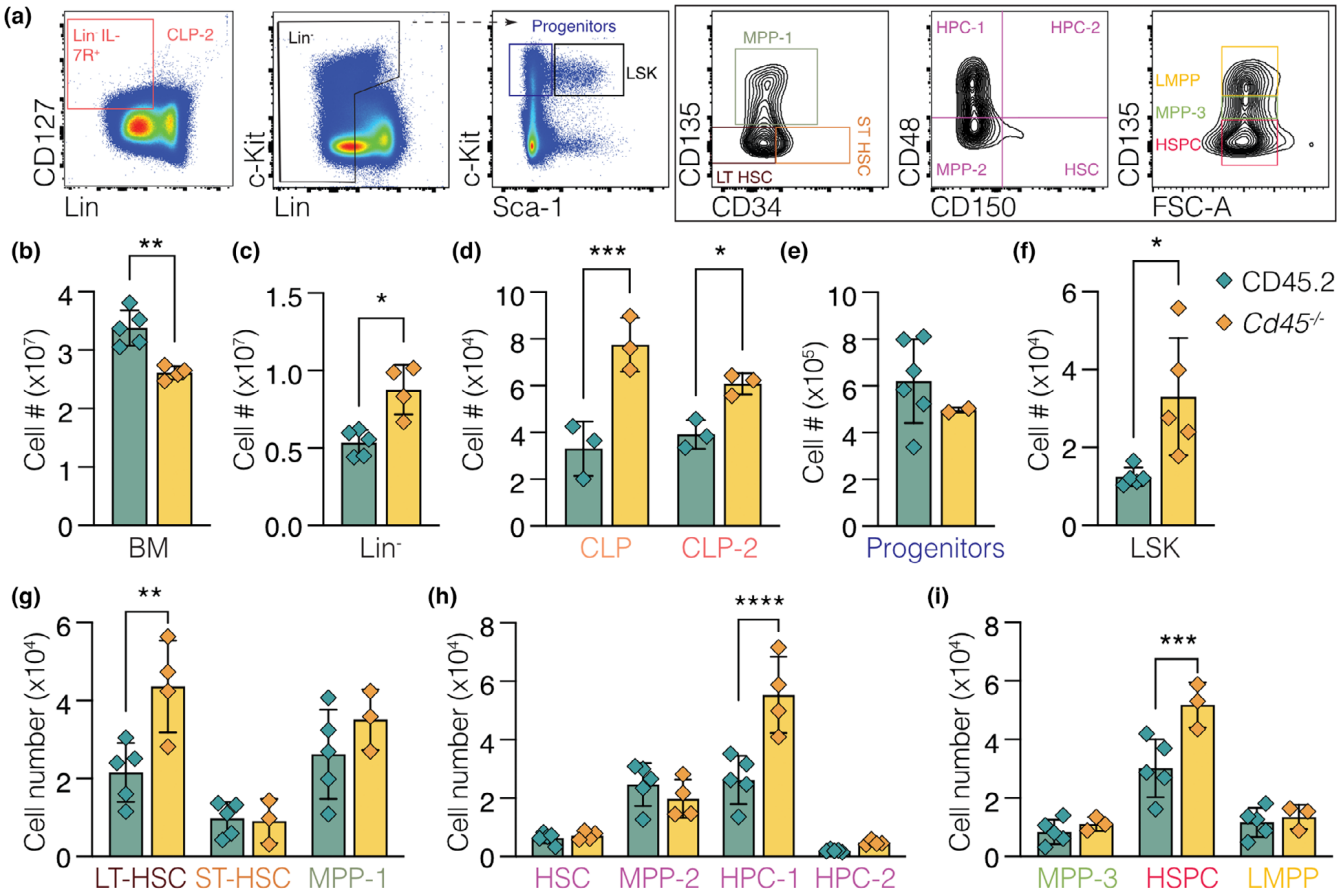
CD45 acts to restrict progenitor and stem cell populations. Bone marrow cells were harvested from 6–8‐week‐old male and female *Cd45* ^*+/+*^ (CD45.2; green) and *Cd45* ^*−/−*^ (yellow) mice for flow cytometric analysis of the HSC compartment. **(a)** Gating strategy to identify populations in the hematopoietic stem cell compartment. Lineage markers (Lin) included CD2, CD4, CD8, Gr1, F4/80, CD19, B220, Ly6G, TER119 and NK1.1. **(b–h)** Enumeration of **(b)** viable bone marrow cells, **(c)** viable lineage negative BM cells, **(d)** the common lymphoid progenitor (CLP) using two recognized gating strategies (CLP: Lin^−^Sca1^med^cKit^med^IL‐7R^+^; and CLP2: Lin^−^FSC^lo^cKit^med^IL‐7R^+^), **(e)** Myeloid/erythroid progenitors (Lin^−^Sca1^−^cKit^+^), **(f)** the LSK (Lin^−^Sca1^+^cKit^+^) population and **(g–i)** subsequent hematopoietic stem cell populations determined by flowcytometry and counting beads (123count eBeads). Representative experiment shown of two independent experiments (*n* = 4 mice of each gender in each experiment; eight in total per genotype). Significance was determined by two‐way ANOVA with Sidak's multiple comparisons test (* < 0.5, ** < 0.01, *** < 0.001 and **** < 0.0001).

Given the increase in CLP cells, the hematopoietic compartment of *Cd45* ^*−/−*^ mice was characterized in greater detail. Lineage negative cells (Lin^−^: CD2^−^CD4^−^ CD8^−^Gr1^−^F4/80^−^CD19^−^B220^−^Ly6G^−^TER119^−^NK1.1^−^) were analyzed for progenitor and stem cells **(**Figure 5a). Again, despite a decrease in the total number of viable bone marrow cells in *Cd45* ^*−/−*^ mice (Figure 5b), the Lin^−^ population was increased **(**Figure 5c). CLPs were characterized by two gating methods (CLP: Lin^−^Sca1^med^cKit^med^IL‐7R^+^; CLP2: Lin^−^FSC^lo^cKit^med^IL‐7R^+^)^38^ and enumerated; both methods confirmed an increased number of CLPs in *Cd45* ^*−/−*^ mice (Figure 5d **)**. No differences in population frequency or enumeration were observed within the myeloid progenitor compartment (Lin^−^Sca1^−^cKit^+^; Figure 5e). The Lin^−^Sca1^+^cKit^+^ (LSK) population, which contains the hematopoietic stem cells, was marginally increased in *Cd45* ^*−/−*^ mice (Figure 5f). A ~2‐fold increase was observed in the following LSK stem cell populations: long‐term hematopoietic stem cells (LT‐HSC; CD41^−^ CD34^−^LSKs), restricted hematopoietic progenitor 1 cells HPC‐1 (CD48^+^CD150^−^LSKs) and hematopoietic stem and progenitor cells (HSPC; CD135^−^FSC^mid^; Figure 5g–i). Although the differences in the HSC compartment of *Cd45* ^*−/−*^ mice appeared modest, they were consistent with CD45 acting to restrict progenitor and stem cell populations.

Collectively, the data suggested that loss of CD45 likely impacted two stages in early hematopoietic development, resulting in a modest increase in LSK/LT‐HSC and CLP populations, potentially contributing to the expansion of the NK cell compartment in *Cd45* ^*−/−*^ mice.

## DISCUSSION

CD45 could be viewed as an immune checkpoint in NK cells, due to the increased NK cell expansion observed in *Cd45* ^*−/−*^ mice.^19^, ^26^ Here we provided evidence that the increased NK cell expansion in mice with a global loss of CD45, stemmed from a defect in an early progenitor/stem cell population, rather than from a specific effect of CD45 on NK cell proliferation or survival. This is supported by the reduced proliferative capacity of *Cd45* ^*−/−*^ NK cells *ex vivo*, the lack of expansion following adoptive transfer of mature *Cd45* ^*−/−*^ NK cell populations into lymphoid deficient mice, and, finally, conditional deletion of CD45 in NK cells, which had no impact on NK cell homeostasis or the ability of transferred NK cells to expand and persist *in vivo*. In addition, we demonstrated that the NK cell hyperplasia in *Cd45* ^*−/−*^ mice was not driven by differential responsiveness to IL‐15, a cytokine required for NK cell survival, proliferation and maturation, nor was differential abundance of systemic IL‐15 in *Cd45* ^*−/−*^ mice a likely cause.

Interestingly, although the NK cell expansion observed in *Cd45* ^*−/−*^ mice was not present in mice with an NK cell specific‐deletion of *Cd45*, the skewing of NK cell maturation towards a mature M2 stage was maintained. Again, this was independent of IL‐15 levels and suggested that CD45 limited NK cell maturation towards an M2 effector stage, *via* a yet unknown mechanism. This observation may also account for the apparent reduction in NK cell proliferation observed *ex vivo*. Mature M2 (CD11b^+^KLRG1^+^) stage NK cells have limited proliferative capacity compared with immature or M1 (CD11b^+^KLRG1^−^) stage NK cells.^26^, ^39^ Given M2 cells predominate in NK cell pools purified from *Cd45* ^*−/−*^ mice, it is perhaps not surprising that we observed significantly reduced *Cd45* ^*−/−*^ NK cell proliferation *in vitro*. Again, this is disconnected from the *in vivo* phenotype where *Cd45* ^*−/−*^ M2 NK cells cycle faster than control M2 NK cells, as determined by BrdU uptake.^26^

The analysis of early progenitor populations revealed a potential role for CD45 in limiting the progenitor/stem pool. Previously, we did not observe a defect in the *Cd45* ^*−/−*^ NK precursor population,^19^ identified as CD122^+^NK1.1^−^DX5^−^CD3^−^CD19^−^ defined by Rosmaraki *et al*.^40^ In the current study, we refined our characterization of the pre‐NKP and rNKP populations using markers described by Carotta *et al*.^41^ and Fathman *et al*.^42^ to reveal a defect within the pre and restricted NK progenitor populations.^41^, ^42^ In addition, we observed an increase within both LSK and CLP *Cd45* ^*−/−*^ populations, which was not captured by hematopoietic analysis of exon 6‐targeted CD45 deficient mice.^19^ However, exon 6‐targeted CD45 deficient mice retain a small thymic population that expresses CD45, suggesting that the lack of perturbation in the lymphoid compartment may be due to residual CD45 expression.^8^

This study attributed the perturbed NK cell homeostasis in CD45‐deficient mice to developmental defects impacting the LT‐HSC and CLP populations. In CD45‐deficient mice, most likely the increase in a common progenitor population, coupled with a faster transit through NK cell development and maturation, manifests as increased numbers of circulating mature NK cells.

Although the systemic use of CD45 inhibitors in the clinic would abrogate TCR and BCR signaling,^23^, ^24^ both of which are required for an effective anti‐cancer immune response, this study identifies a potential clinical application in adoptive immunotherapy. Here we investigated the role that CD45 plays in regulating expression of NKp46, resulting in increased NK cell numbers, predicting that inhibition of CD45 or deletion of the *ptrprc/Cd45* gene could enhance the yield of NK cells derived from CD34^+^ umbilical cord blood cells or inducible pluripotent stem cells. Improving the yield of NK cells would help to address one of the technical limitations currently associated with this approach.

While it is clear that deleting *Cd45* will enhance the NK cell differentiation, this study reveals that CD45 is important for proper mature NK cell function. Indeed, other studies (e.g. Hesslein *et al*.^20^) have shown impaired cytotoxic responses by CD45‐deficient murine NK cells. Therefore, an optimal translational intervention would seek to impair CD45 function during cell development/differentiation, and then reactivate it in mature NK cells. Critically, further work is needed to confirm that these observations translate to human NK cell development and do not negatively impact NK cell cytotoxicity.

## METHODS

### Mice

All animal experiments followed the National Health and Medical Research Council (NHMRC) Code of Practice for the Care and Use of Animals for Scientific Purposes guidelines and were conducted in accordance with the regulatory standards approved by the Walter & Eliza Hall Animal Ethics Committee (AEC2019.034, AEC2018.040, AEC2021.011 and SABC) and Monash University Animal Ethics Committee (25 004, 22 111). All strains (Table 1) were maintained on a C57BL/6 background and bred at either the Walter & Eliza Hall Institute or Monash University.

**Table 1:** Genetically modified mouse strains

Strain^a^	AKA^b^	Description	Reference^c^
Cd45tm1Tyb	*Cd45* ^*−/−*^	Disruption of *Cd45* gene by insertion of a neomycin gene	10
Mcl‐1^*fl*/*fl*^ Ncr1‐Cre	*Mcl1* ^*fl*/*fl*^ *Ncr1* ^*iCre*/+^	NK lymphopenic mice	36
IL‐15(‐/‐)	IL‐15^−/−^	C57BL/6 mice genetically deficient in interleukin 15	43
IL‐15tg	IL‐15 Tg^T/+^	IL‐15 transgene where three posttranscriptional checkpoints were eliminated. Transgene overexpression achieved by the MHC class I promoter	44
B6 Cd45.1	Ly5.1/J	C57BL/6 congenic strain carrying the Ptprca pan leukocyte marker commonly known as CD45.1 or Ly5.1	45
C57BL/6J	C57BL/6	Acquired from JAX in 1989	46
Rag2^−/−^common gamma chain^−/−^	*Rag2* ^*−/−*^ γ *c* ^*−/−*^	Completely alymphoid (T‐, B‐, NK‐) mice	47
B6.129S7‐Rag1<tm1Mom>	*Rag1*	Mice homozygous for the Rag1tm1Mom mutation produce no mature T cells or B cells	48
Ptprc^*fl*/*fl*^	*Cd45* ^*fl*/*fl*^	Generated by Monash Genome Modification Platform	This publication
Nkp46^*iCre*^/wt	*Ncr1* ^*iCre*/+^	“Knock‐in” mice containing the iCre recombinase gene under control of the Ncr1 gene promoter	37
Ptprc^*fl*/*fl*^ Ncr1^*Cre*/+^	*Cd45* ^*fl*/*fl*^ *Ncr1* ^*Ki*/+^	Mice with conditional deletion of CD45 in NKp46 expressing cells	This publication

^a^ Strain name as it appeared in first publication.

^b^ Common strain name.

^c^ Strain generation and characterization.

### Generation of mice with conditional deletion of *Cd45* (*Ptprc*) in NK cells

Mice with a floxed Cd45 allele (*Cd45* ^*fl/+*^) were generated on a C57BL/6J background by the Monash Genome Modification Platform and the Australian Phenomics Network, schematic of generation provided in Supplementary figure 1. Clustered Regularly Interspaced Short Palindromic Repeats (CRISPR) technology was used to generate mice carrying *Cd45* conditional allele (CKO). In short, CRISPR ribonucleoproteins (RNPs; Cas9 protein in complex with sgRNAs targeting sequences flanking *Cd45* exon 14) and a ssDNA donor repair template (floxed Exon14 with homology arms) were microinjected into fertilized one‐cell stage embryos. The integration site was confirmed using 5′ and 3′ long range PCR, and the number of integrations was determined using droplet digital PCR (targeting Exon14), confirming two copies of Exon14 in heterozygous mice. sgRNA guide, donor template and PCR primer sequences are provided in Supplementary table 1. Cre‐lox deletion of exon 14 results in a premature stop codon in exon 15. Furthermore, translation of the compromised mRNA sequence predicts a truncated Cd45 protein, which lacks a transmembrane and cytoplasmic domain and is expected to be non‐functional. Established *Cd45* ^*fl/fl*^ mice were crossed with *Ncr1* ^*iCre/+*^ mice to conditionally delete *Cd45* from mature NK cells (*Cd45* ^*fl/fl*^ *Ncr1* ^*iCre/+*^).^37^ These strains were maintained on a C57BL/6 background and bred at Monash University animal facilities (Clayton).

### Organ processing

Blood, BM, lungs, thymus and spleen were collected from 6–8‐week‐old mice, each organ was processed to a single cell suspension for further analysis as follows.

#### Blood

Retro‐orbital, mandibular or cardiac bleeds were collected in Microvette® 500 K3 EDTA tubes (Sarstedt, Nümbrecht, Germany), transferred to a 5‐mL polypropylene Falcon tube (Corning, New York, NY, USA), and adjusted to a total volume of 1 mL with phosphate‐buffered saline (PBS; Gibco, New York, NY, USA). The cell suspension was under‐layered with 2 mL of Histopaque‐1077 (Sigma Aldrich, St Louis, MO, USA) and centrifuged at 300 *g* for 15 min at room temperature (RT). Leukocytes can be found at the interface layer, these were then transferred to a 10‐mL Falcon tube, washed twice with ice‐cold PBS and resuspended in FACS buffer [PBS, 2% FBS (Bovogen Biologicals, Lot#2009A; Clayton, VIC, Australia), 1 mm ethylenediaminetetraacetic acid (EDTA; Invitrogen™, MA, USA)]. Enriched leukocytes were used in subsequent experiments.

#### Bone marrow

Unless otherwise specified, femurs and tibias were collected, and BM flushed into a 10‐mL Falcon tube using a PBS filled syringe. Cell suspensions were passed through a 70‐μm cell strainer and centrifuged at 300 *g* for 5 min at 4°C. The cell pellet was resuspended in 1 mL of red cell removal buffer [RCRB; 156 mm NH_4_Cl (Sigma Aldrich), 11.9 mm, NaHCO_3_ (Sigma Aldrich) and 0.097 mm, EDTA] and incubated for 5 min at RT. The cells were washed twice with ice‐cold PBS and resuspended in FACS buffer. BM single cell suspensions were used in subsequent experiments.

#### Lungs

Lungs were minced at RT into small fragments. Minced lungs were thoroughly mixed with 5 mL digestion buffer (1 mg mL^−1^ collagenase IV and 30 μg mL^−1^ DNase I in PBS) and incubated for 30 min at 37°C. To further dissociate cells, the digested tissue was forcefully passed through a 70‐μm cell strainer with PBS, and the cell suspension was centrifuged at 300 *g* for 5 min at 4°C. The cell pellet was resuspended in 5 mL of RCRB, incubated for 5 min at RT and then washed twice with ice‐cold PBS.

#### Thymi

Thymi were forcefully passed through a 70‐μm cell strainer with PBS, collected in a 10‐mL Falcon tube and centrifuged at 300 *g* for 5 min at 4°C. The cell pellet was resuspended in 5 mL of RCRB, incubated for 5 min at RT and then washed twice with ice‐cold PBS.

#### Spleens

Spleens were forcefully passed through a 70‐μm cell strainer with PBS, collected in a 10‐mL Falcon tube and centrifuged at 300 *g* for 5 min at 4°C. The cell pellet was resuspended in 1 mL of PBS and passed through a 70‐μm cell strainer with PBS, adjusting the volume to 10 mL in a 10‐mL Falcon collection tube, prior to centrifugation at 300 *g* for 5 min at 4°C.

### Flow cytometry analysis

Cell proliferation and intracellular IFNγ data were collected on a FACSVerse (BD Biosciences, Franklin Lakes, NJ, USA) using BD FACSuite software. NK cell progenitor data were collected on a FACSymphony (BD Biosciences) using BD FACS Diva software. Data for all other experiments were collected on a BD LSR Fortessa X‐20 using BD FACS Diva software. All analysis and statistics were performed using FlowJo v10 software and Prism GraphPad, respectively.

Flow cytometric analysis of viable cells was performed by excluding debris (FSC‐A; Forward Scatter‐Area low events), doublets (FSC‐A *versus* FSC‐H; Forward Scatter‐Height) and dead cells (dead‐cell indicator dye negative). Cell subsets were gated based on surface marker expression (Table 2).

**Table 2:** Markers used to gate various immune populations by flow cytometry.

Cell subset	Markers used for gating
Leukocytes	CD45^+^
Pan T cells	CD45^+^ CD3^+^
NK cells	CD45^+^ CD3^−^ TCRβ^−^ NK1.1^+^ NKp46^+^
Lineage^neg^	CD3e^−^ CD8^−^ CD4^−^ CD19^−^ TER119^−^ Ly6G^−^ CD11b^−^ Ly6D^−^ NK1.1^−^
CLP^a^ (defined by Carotta *et al*.^41^)	Lineage^neg^ 2B4^+^ CD27^+^ cKit^−^ CD127^+^ Flt3^+^
Pre‐NKP	Lineage^neg^ 2B4^+^ CD27^+^ cKit^−^ CD127^+^ Flt3^−^ CD122^−^
rNKP	Lineage^neg^ 2B4^+^ CD27^+^ cKit^−^ CD127^+^ Flt3^−^ CD122^+^
Lin^−^	CD2^−^ CD4^−^ CD8^−^ Gr1^−^ F4/80^−^ CD19^−^ B220^−^ Ly6G^−^ TER119^−^ NK1.1^−^
CLP1	Lin^−^ Sca1^med^ cKit^med^ IL‐7R^+^
CLP2	Lin^−^ FSC^lo^ cKit^med^ IL‐7R^+^
Myeloid progenitor compartment	Lin^−^ Sca1^−^ cKit^+^
LSK	Lin^−^ Sca1^+^ cKit^+^
LT‐HSC	CD135^−^ CD34^−^ LSK
ST‐HSC	CD135^−^ CD34^+^ LSK
MPP‐1	CD135^+^ CD34^−^ LSK
HPC‐1	CD48^+^ CD150^−^ LSK
HPC‐2	CD48^+^ CD150^+^ LSK
MPP‐2	CD48^−^ CD150^−^ LSK
HSC	CD48^−^ CD150^+^ LSK
MPP‐3	CD135^med^ FSC^med^ LSK
LMPP	CD135^hi^ FSC^med^ LSK
HSPC	CD135^−^ FSC^med^ LSK

^a^ Flt3 is also known as Flk2, Flk2/3 and CD135; CD127 is also known as IL‐7R.

### Production of IFNγ by splenic NK cells

Spleens were processed and intracellular IFNγ measured as previously described.^49^ In short, splenocytes of 6–8‐week‐old mice were stimulated with 20 ng mL^−1^ recombinant mouse IL‐12 (Peprotech, Rehovot, Israel) in combination with 200 ng mL^−1^ recombinant mouse IL‐18 (Peprotech) or 50 ng mL^−1^ of recombinant human IL‐15 (Miltenyi Biotec, Bergisch Gladbach, Germany) for 4 h prior to fixation/permeabilization (Foxp3/Transcription Factor Staining Buffer Set; eBioscience™, San Diego, CA, USA) for detection of intracellular IFNγ by flow cytometry.

### NK cell purification

Splenic NK cells from 6–8‐week‐old adult mice were purified using Miltenyi Biotec's NK Cell isolation kit mouse (130‐15‐818) as per the manufacturer's instructions.

### *In vitro* proliferation assays

NK cell proliferation studies, including cohort number and mean division number determination, were performed as described,^35^ and based on previously published methods using T cells.^50^ In short, purified NK cells are incubated with 0.1 nm of CellTrace Violet (CTV; Invitrogen™) in phosphate buffered saline (PBS) supplemented with 0.1% bovine serum albumin (Sigma Aldrich) for 20 min at 37°C. Labeled cells were subsequently washed twice with ice‐cold NK complete media [IMDM (Gibco) containing 10% v/v heat‐inactivated FBS, 1% v/v penicillin–streptomycin, 1× GlutaMAX™ (Gibco), 55 μm β‐mercaptoethanol (Sigma Aldrich)] to quench the labeling reaction. Labeled NK cells (4000–10 000 per well) were seeded into 96‐well round‐bottom plates and cultured at 37°C in 5% CO_2_. A mixture of propidium iodide (PI; 200 nm; Sigma Aldrich) and 123count eBeadsTM (5005 beads/well; Invitrogen™) was added to cultures prior to flow cytometric analysis. Cells were analyzed daily by flow cytometry.

### Generation of bone marrow chimeras

Bone marrow chimeras were generated by lethally irradiating (2 × 550 rads) host mice and reconstituting by I.V. injection into the tail vein with 6 × 10^6^ donor BM cells for 100% chimeras, or 3 × 10^6^ control donor BM and 3 × 10^6^ experimental donor BM for mixed chimeras. Generally, host and donor mice express allelic variants of the pan‐hematopoietic cell marker CD45, also known as CD45.1 (Ly5.1) and CD45.2 (Ly5.2). Donor BM was collected from femur, tibia, pelvis, radius ulna, humerus and cervical vertebrae by crushing the bones in a mortar with a pestle and PBS. BM suspension was passed through a 70‐μm cell strainer, centrifuged at 300 *g* for 5 min at 4°C, resuspended in PBS and the cells counted, prior to injection. Immediately post‐irradiation, neomycin treated water (2 mg mL^−1^ neomycin sulfate in drinking water; Sigma Aldrich) was made available to mice for up to 3 weeks. Reconstitution of the hematopoietic compartment was checked at 6‐week post‐bone marrow transplantation. Peripheral blood was obtained by retro‐orbital bleeding using a sterile hematocrit capillary tube (Sarstedt). Post‐processing, blood samples were stained with anti‐CD45.1 and anti‐CD45.2 for flow cytometric analysis of the leukocyte compartment.

### Adoptive transfer of mouse NK cells

Splenic NK cells from 6–8‐week‐old mice were enriched from single cell suspensions by negative depletion. Splenocytes were labeled with biotinylated antibody cocktail (CD3, CD4, CD8, MHC‐II, Ly6G, F4/80, TER119 and CD19; antibody details in Supplementary table 2) followed by enrichment using MagniSort™ streptavidin negative selection beads (Invitrogen™). NK cells enriched from C57BL/6 (CD45.2) and *Cd45* ^*−/−*^ mice (~70% purity) were labeled with 0.1 nm CTV. A portion of the cells were labeled with fluorescently conjugated antibodies against NK cell markers in combination with a mixture of PI (200 nm) and 123count eBeadsTM (5005 beads/well; Invitrogen™) prior to flow cytometric analysis. NK cells were counted, mixed (1:1) and injected into the tail vein of host mice.

### Statistical analyses

Two‐way ANOVA with Sidak's multiple comparisons test (GraphPad Prism 9) was used to compare groups for all experiments. Significance was determined to be *P ≤ 0.05, **P ≤ 0.01, ***P ≤ 0.001 and ****P ≤ 0.0001.

## CONFLICT OF INTEREST

NDH and JR are founders and shareholders in oNKo‐Innate. NDH and SEN are inventors on a patent relating to adoptive NK cell therapy that has been licensed by ONK Therapeutics and receive royalties. NDH serves on an advisory board for Bristol Myers Squibb. The authors declare no competing interests.

## AUTHOR CONTRIBUTIONS

LGMG designed and executed experiments, and co‐wrote the manuscript; CDH designed and performed progenitor analyses; GMB, EL and ZS performed experiments; WG helped design and supervise early experiments; JR designed and supervised the study; JEV supervised the study; SEN supervised the study and co‐wrote the manuscript; NDH initiated, designed and supervised the study; all authors reviewed and approved the manuscript.

## Supporting information


Supporting Information

## Data Availability

All data generated from this study, if not included in this article, are available from the corresponding authors on reasonable request.
