# Supplementary material for: CD45 limits early Natural Killer cell development
Source: Immunol Cell Biol. 2023 Oct 19;102(1):58–70. doi: 10.1111/imcb.12701 (PMC10952700; doi:10.1111/imcb.12701)
Supplement: Supplementary file 1 — Supporting Information [file IMCB-102-58-s001.docx]

**CD45 limits early Natural Killer cell development**

Lizeth G Meza Guzman^1,2^, Craig D Hyland^2^, Grace M Bidgood^1,2^, Evelyn Leong^1^, Zihan Shen^3^, Wilford Goh^2^, Jai Rautela^3^, James E Vince^2^, Sandra E Nicholson^1,2,#^, Nicholas D Huntington^3,#^

Supporting information

***
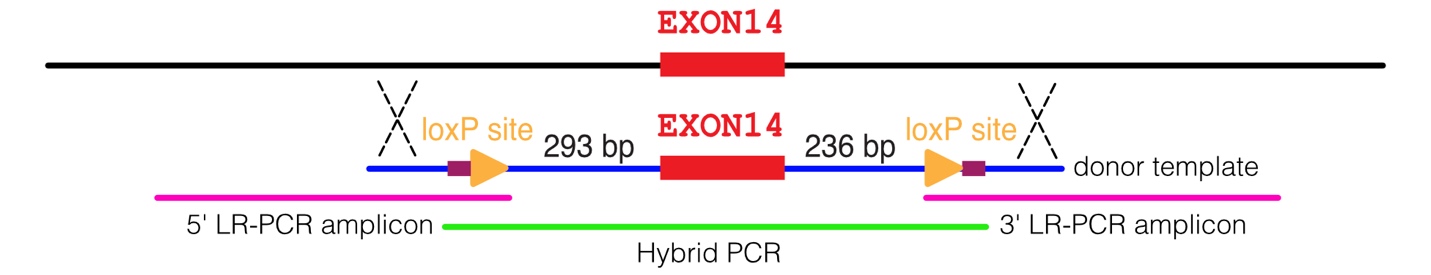
***

**Supplementary figure 1. Generation of mice carrying the conditional ptprc allele.** Schematic of modified Cd45 allele. Black line: Genomic area containing exon 14 (red) selected for modification. Blue line: Donor template showing location of introduced loxP sites (yellow) flanking exon 14 (red). Pink lines: 5’ and 3’ long range (LR) polymerase chain reaction (PCR) amplicons (pink) used to confirm correct integration of donor template. Green line: Hybrid PCR amplicon used to verify that both loxP sites are located on the same allele. Sequences of guides, primers and donor template are provided in **Supplementary table 1**.

**
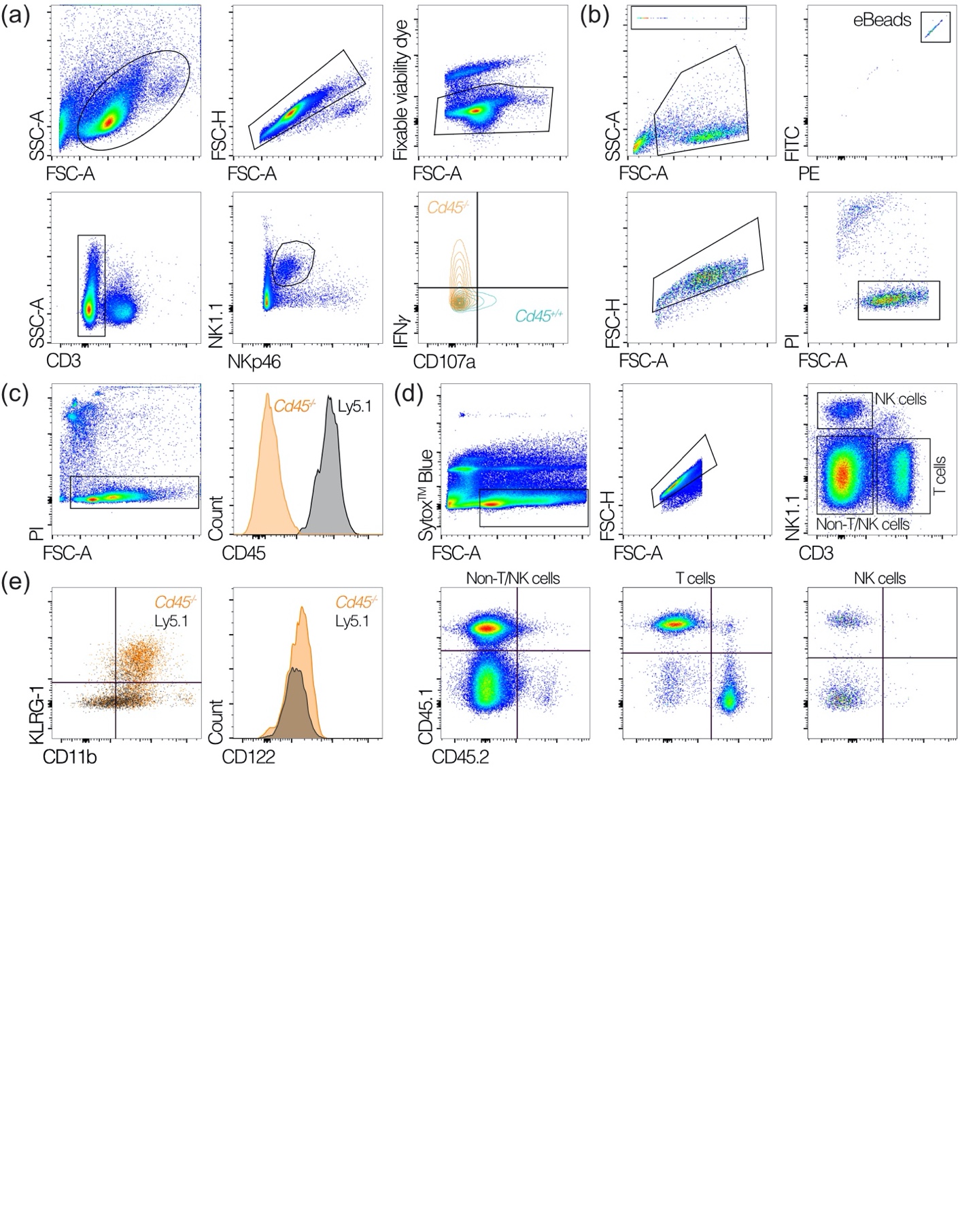
**

**Supplementary figure 2. Representative gating strategy corresponding to data in Figure 1. (a)** Splenocytes from either control C57BL/6 mice (CD45.2; green) or Cd45^-/-^ (yellow) 6-8-week-old mice were gated to exclude debris (FSC-A^lo^; Forward Scatter-Area low events), doublets (FSC-A vs FSC-H; Forward Scatter-Height), dead cells (Fixable viability dye^-^), and T cells (CD3^-^TCRß^-^), prior to identification of NK cells based on expression of NK1.1 and NKp46 (NK1.1^+^NKp46^+^) (corresponding to **Figure 1 a**). NK cells were further analysed for expression IFN$\gamma$. **(b)** Representative gating for *in vitro* proliferation on day 8 (corresponding to **Figure 1 b-d**). Viable NK cells were gated to exclude debris (FSC-A^lo^), doublets (FSC-A vs FSC-H) and dead cells (PI^-^). The event counts of gated NK cells and eBeads^TM^ [FSC-A^lo^ and Side Scatter-Area high events (SSC-A^hi^)] were exported from FlowJo^TM^ for further analysis. **(c-e)** BM chimeras in **Figure 1 e-j. (c)** Single cell suspensions of viable bone marrow (PI^-^) from 6-8-week-old donor mice C57BL/6 (CD45.1; Ly5.1; grey; CD45^+^) and *Cd45^-/-^* (yellow, CD45^-^) mice, were mixed in a 1:1 ratio prior to transplantation into lethally irradiated 6-8-week-old host mice (corresponding to **Figure 1 e-j**). **(d)** Reconstitution of BM chimeras in the immune compartment of the spleen 8-weeks post transplantation (IL-15^-/-^ host used as example). Viable (SytoxBlue^-^), single (FSC-A vs FSC-H) cells gated prior to gating of non-T/NK cells (CD3^-^NK1.1^-^), pan T cells (CD3^+^), and NK cells (CD3^-^/NK1.1^+^/Nk46^+^). Populations were further gated on CD45^-^, CD45.1^+^ and Cd45.2^+^. **(e)** NK maturation profile for M1 (KLRG1^-^CD11b^+^) and M2 (KLRG1^+^CD11b^+^) NK cells (corresponding to **Figure 1i**) and surface expression of CD122 (corresponding to **Figure 1j)**.


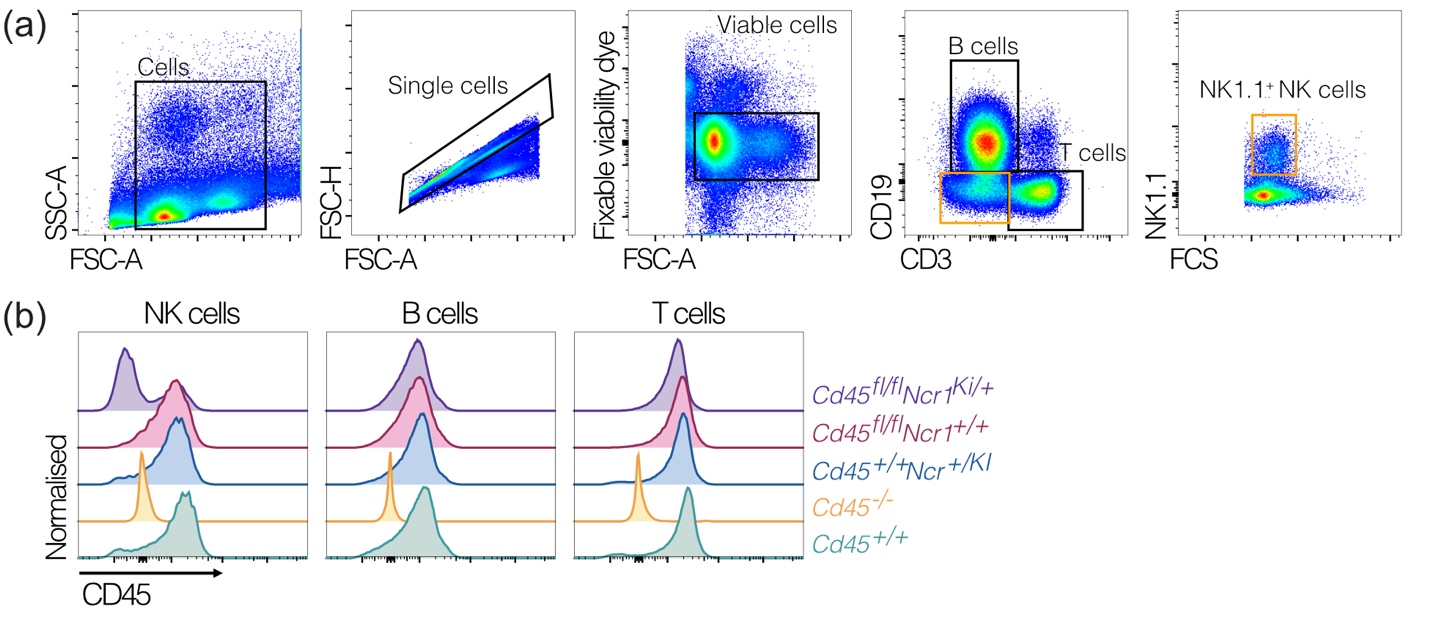


**Supplementary figure 3. Surface expression of CD45 on lymphocytes.** Splenocytes from 6-10-week-old male and female control C57BL/6 (*Cd45^+/+^*; green), Cd45^-/-^ (yellow), Cd45^+/+^Ncr1^iCre/+^ (blue), Cd45^fl/fl^Ncr1^+/+^ (pink), and Cd45^fl/fl^Ncr1^iCre/+^ (purple) mice were analyzed by flow cytometry for expression of CD45 on NK, B and T cells. **(a)** Representative gating for NK (CD19^-^CD3^-^NK1.1^+^), B (CD19^+^CD3^-^), and T (CD19^-^CD3^+^) cells, initially gated to exclude debris (FSC-A^lo^; Forward Scatter-Area low events), doublets (FSC-A vs FSC-H; Forward Scatter-Height) and dead cells (Fixable viability dye^-^). **(b)** Histograms showing CD45 surface expression in NK, B and T cells (representative of n = 4 mice per genotype).


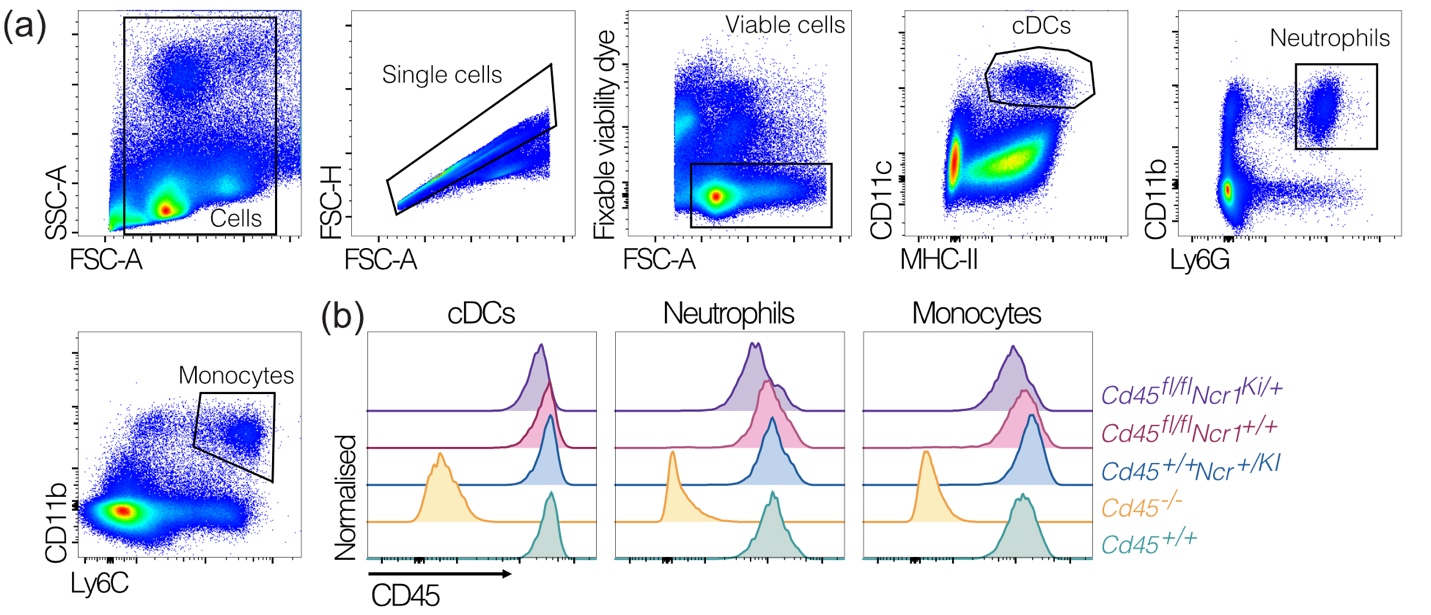


**Supplementary figure 4. Surface expression of CD45 on myeloid cells.** Splenocytes from 6-10-week-old male and female control C57BL/6 (*Cd45^+/+^*; green), Cd45^-/-^ (yellow), Cd45^+/+^Ncr1^iCre/+^ (blue), Cd45^fl/fl^Ncr1^+/+^ (pink), and Cd45^fl/fl^Ncr1^iCre/+^ (purple) mice were analyzed by flow cytometry for expression of CD45 on myeloid cells. **(a)** Representative gating for conventional dendritic cells (cDCs; CD11c^+^MHC-II^+^), neutrophils (cDCs^-^CD11b^+^Ly6G^+^) and monocytes (cDCs^-^neutrohil^-^CD11b^+^Ly6C^+^), initially gated to exclude debris (FSC-A^lo^; Forward Scatter-Area low events), doublets (FSC-A vs FSC-H; Forward Scatter-Height) and dead cells (Fixable viability dye^-^). **(b)** Histograms showing CD45 expression on cDCs, neutrophils and monocytes (representative of n = 4 mice per genotype)­.


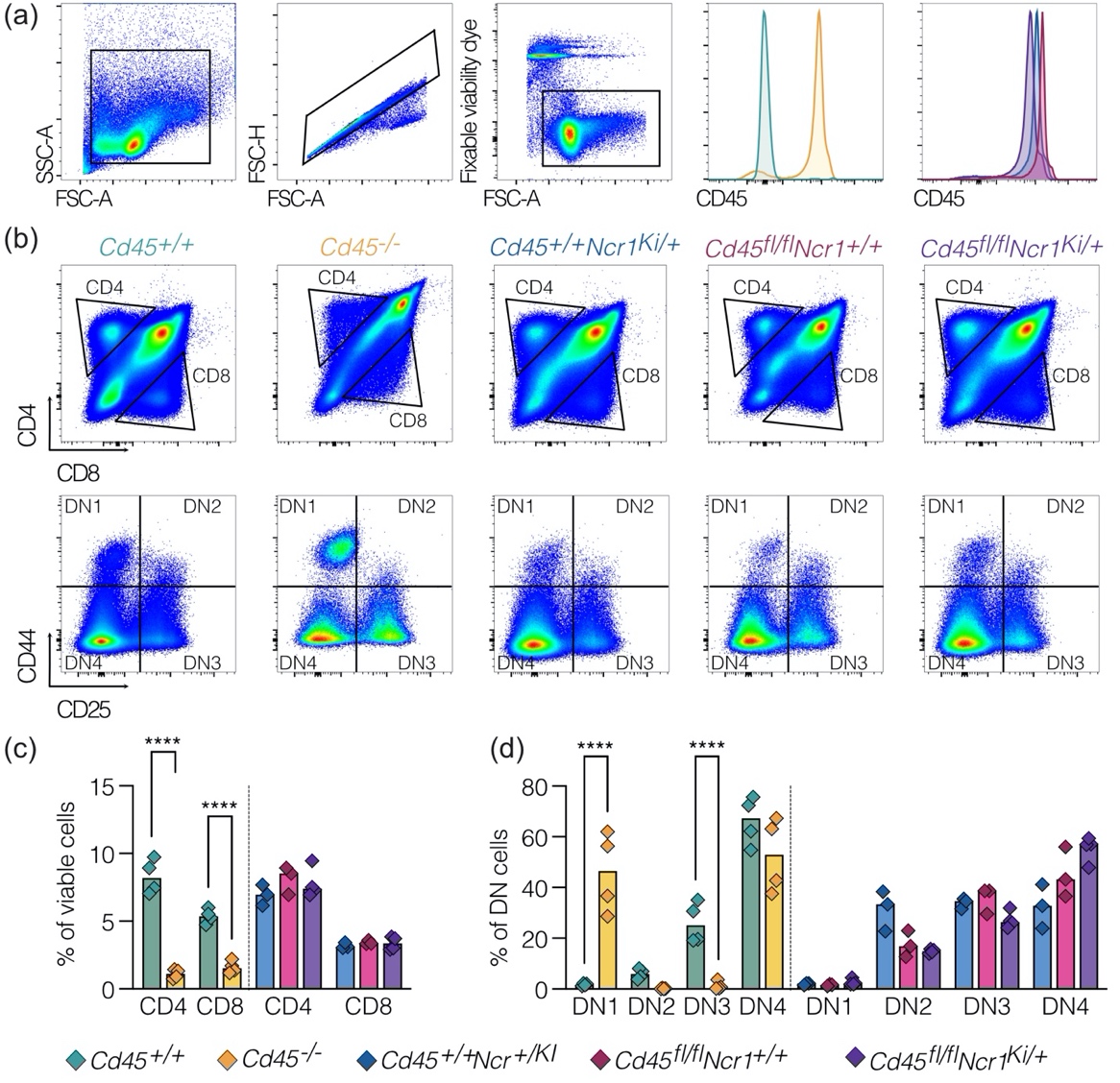


**Supplementary figure 5. Development of T cells in *Cd45^fl/fl^Ncr1^+/ki^* mice.** Thymocytes from 6-10-week-old male and female control C57BL/6 (*Cd45^+/+^*; green), Cd45^-/-^ (yellow), Cd45^+/+^Ncr1^iCre/+^ (blue), Cd45^fl/fl^Ncr1^+/+^ (pink), and Cd45^fl/fl^Ncr1^iCre/+^ (purple) mice were analyzed by flow cytometry for expression of CD45 and T cell development. **(a)** Thymocytes were gated to exclude debris (FSC-A^lo^; Forward Scatter-Area low events), doublets (FSC-A vs FSC-H; Forward Scatter-Height) and dead cells (Fixable viability dye^-^) and analysed for *Cd45* expression. Histograms show CD45 surface expression on viable thymocytes (right panels, representative of n = 4 mice per genotype). **(b)** Example gating of CD4^+^ and CD8^+^ T cells from viable thymocytes and showing developmental stages (DN1-4) within double negative T cells (CD4^-^CD8^-^). **(c)** Percentages of viable CD4^+^ and CD8^+^ T cells from thymus. CD4^+^ and CD8^+^ T cells are reduced in mice with global (but not NK conditional) deletion of Cd45. **(d)** Percentages of viable cells in developmental stages (DN1-4) from double negative T cells (CD4^-^CD8^-^).

**
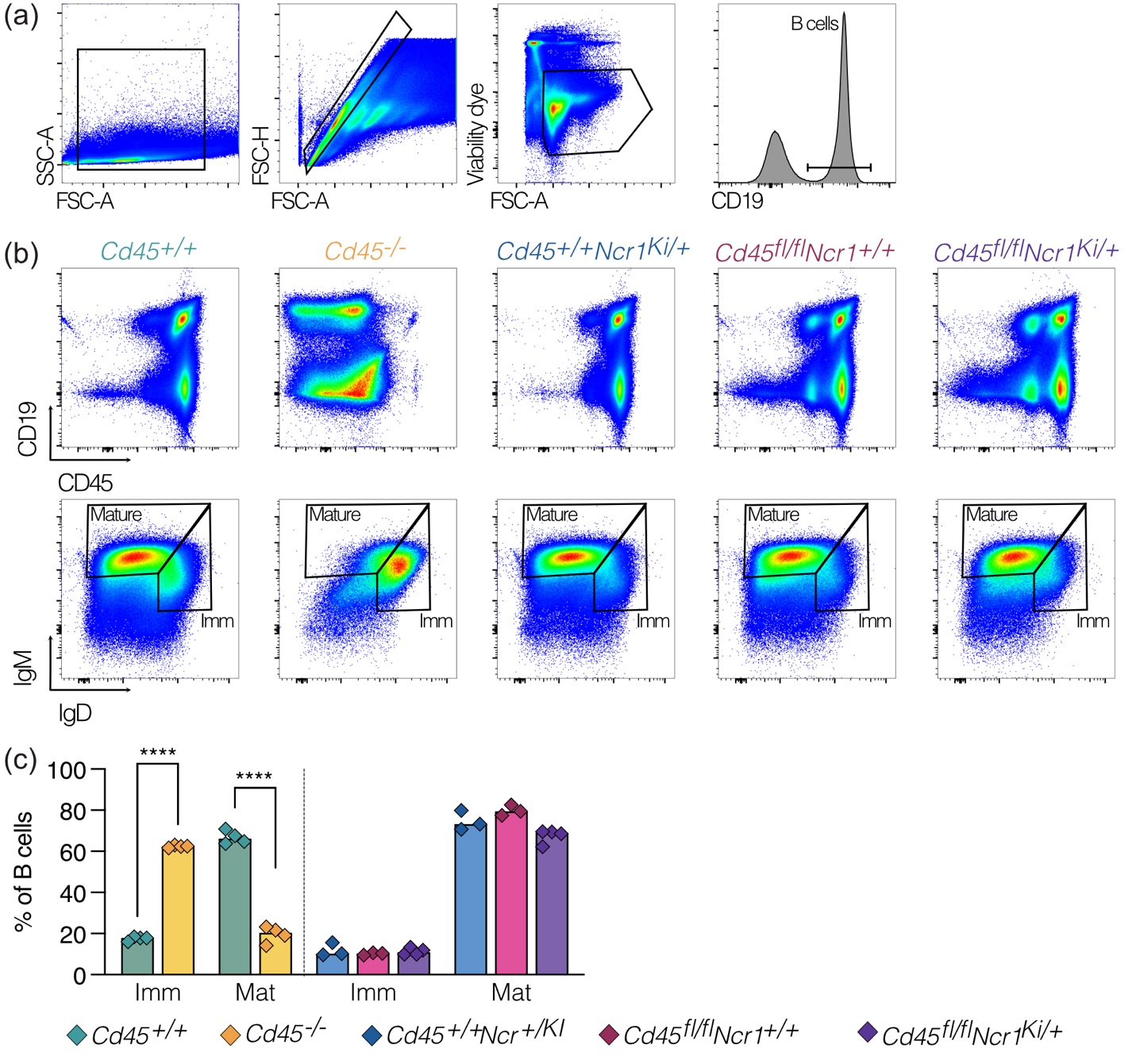
 Supplementary figure 6. Development of B cells in *Cd45^fl/fl^Ncr1^+/ki^* mice.** Splenocytes from 6-10-week-old male and female control C57BL/6 (*Cd45^+/+^*; green), Cd45^-/-^ (yellow), Cd45^+/+^Ncr1^iCre/+^ (blue), Cd45^fl/fl^Ncr1^+/+^ (pink), and Cd45^fl/fl^Ncr1^iCre/+^ (purple) mice were analysed by flow cytometry for expression of CD45 and B cell development. **(a)** B cells were gated to exclude debris (FSC-A^lo^; Forward Scatter-Area low events), doublets (FSC-A vs FSC-H; Forward Scatter-Height), and dead cells (Fixable viability dye^-^), and analysed for expression of CD19 (histogram). **(b)** Example gating showing CD19 and CD45 expression on viable cells, and B cell maturation profile (IgM and IgD) of viable CD19^+^ B cells. **(c)** Percentages of immature (Imm; IgD^+^IgM^+^) and mature (IgD^-^IgM^+^) B cells.


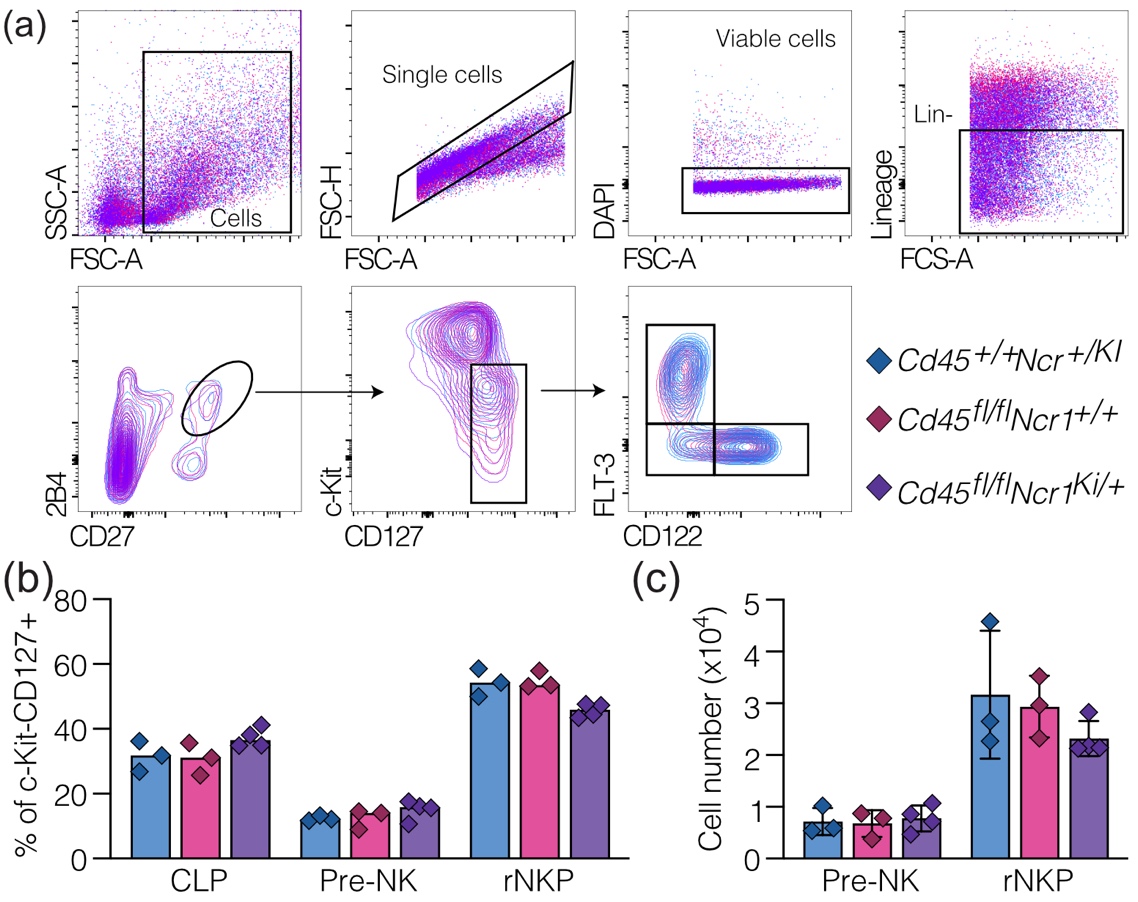


**Supplementary figure 7. Development of NK cells in *Cd45^fl/fl^Ncr1^+/ki^* mice.** Bone marrow cells from 6-10-week-old male and female Cd45^+/+^Ncr1^iCre/+^ (blue), Cd45^fl/fl^Ncr1^+/+^ (pink), and Cd45^fl/fl^Ncr1^iCre/+^ (purple) mice was analysed by flow cytometry for NK cell development. **(a)** Gating strategy for analysis of NK progenitors post-exclusion of other lineages (CD3e, CD8, CD4, CD19, TER119, Ly6G, CD11b, Ly6D and NK1.1). NK cell populations were analysed based on expression of 2B4, CD27, cKit and CD127. FLT3 expression identifies the common lymphoid progenitor (CLP; CD3e^-^CD8^-^CD4^-^CD19^-^TER119^-^Ly6G^-^CD11b^-^Ly6D^-^NK1.1^-^; 2B4^+^CD27^+^; cKit^-^CD127^+^; Flt3^+^), while lack of FLT3 expression defines NK progenitors (NKP: CD3e^-^CD8^-^CD4^-^CD19^-^TER119^-^Ly6G^-^CD11b^-^Ly6D^-^NK1.1^-^; 2B4^+^CD27^+^; cKit^-^CD127^+^; Flt3^‑^). Two NK progenitor populations have been defined, the pre-NKP (NKP; CD122^-^) and restricted NKP (rNKP: NKP; CD122^+^). **(b)** Frequencies of CLP, pre-NKP and rNKP populations. **(c)** Absolute cell numbers.

**Supplementary table 1. Sequences used to generate floxed Cd45 allele (*Cd45^fllWT^*) mice.**

| CRISPR guides | Sequence |
| --- | --- |
| sgRNA1 5’ | AACAGTTAAGTTCTCCTGTA |
| sgRNA2 5’ | TCAAAATGACTTAACTGTAG |
|  |  |
| 5’ long range PCR |  |
| Forward 5’ | TAGCTTGATGGGGAAAGAAGAAGC |
| Reverse 5’ | CGAAGTTATGAGCTCCATGGCCTGT |
|  |  |
| 3’ long range PCR |  |
| Forward 5’ | CGAAGTTATGCATGCGAATTCCTGT |
| Reverse 5’ | AGACATAGGTCCCAAATGTTGTTT |
|  |  |
| Hybrid PCR |  |
| Forward 5’ | CGTACAGGCCATGGAGCTCATAA |
| Reverse 5’ | GTTTCCCCTACAGGAATTCGCA |
|  |  |
| Digital droplet PCR |  |
| ddPCR up | TCTTTATTTTGATAGGTCCGGACA |
| ddPCR PROBE | /56-FAM/TGGAATGAA/ZEN/AACCTCCCGGCCGACAGA/3IABkFQ/ |
| ddPCR down | CCTCCACTTCTGACTACCAAAA |
|  |  |
| donor template^1^ | GTAAGACATTCTCAAAGAATGAATGAAAATATTGGAAAATTTCTAAGTCCTACAGCCATATGCTCTTGCCTTTTCAGGTTTCTGGGATATTAAAACCAAGGAAAGTGATTAGTTGCTTGAAAAAAAATCTCACTTGGTGTTGAGTAGAATAAATAGGCACAAGAGAGGAGGAGGGCTTGGGCCGTACAGGCCATGGAGCTCATAACTTCGTATAGCATACATTATACGAAGTTATAGAACTTAACTGTTTCTCAAGCATCCTTCCCGGTCCCCATCCTTCTCACTTCTCACTTCTTCTCACTGCTATTTCATTCCACGTCCTGTTGTTTAGAGATTGTGCACATACTTAGCGCTTCAAGCATGTCTTCTGTGTTAATAATGAATAAGTGATTTTAAAATAATTTTGAATCTGTGTTTGTGTGATGCAGATCAAAGTTGAGAATGCTTTACATTGTACTCATGCTTCAAGGTATTTAAACTTTTACATGTCAAAATATTAAGATAACAAATGTC**TCTTTATTTTGATAGGTCCGGACAAGGTCAATGGAATGAAAACCTCCCGGCCGACAGACAATAGTATAAATGTTACATGTGGTCCTCCTTATGAAACTAATGGCCCTAAAACCTTTTACATTTTGGTAGTCAGAAGTGGAGG**TTCTTTTGTTACAAAATACAACAAGACAAACTGTCAGTTTTATGTAGATAATCTCTACTATTCAACTGACTATGAGTTTCTGGTAAGGTCATGTGTTCCTTATTCACAGTCTTCCCGAGATATATTATAGTGTTTCATACTTAGTACTTGCATGGCATTAATGATAGTGCTCACATGGACTTACGGATCTCTGATGACTCGTGAAATCAATGATTTAGAACATCAGCGATTTGATGCCAAGTGTTTTCTCTCTGGATTGAAAAGCAATTCTAAATGGCAAACCTTTCTCAGGATATTTGCTACTTCAAAATGACTTAAATAACTTCGTATAGCATACATTATACGAAGTTATGCATGCGAATTCCTGTAGGGGAAACATCGTTCATGTGCCTGTGAGGAGGCATTTCCTGAGCAGTGGATGTTCACTTTCCGTATTTATTTTCACTAAGTTTTCAACTACAAAATCTTAGGTGTGCTTTGAGGTTAGAAAATACTAAAACAAATTAAAAAAAAACATTTGAACATAAAGAACCTTAAGATACCTATTATTTCACTGTGAA |
|  |  |
| Predicted truncated CD45 protein | MTMGLWLKLLAFGFALLDTEVFVTGQTPTPSDELSTTENALLLPQSDPLPARTTESTPPSISERGNGSSETTYHPGVLSTLLPHLSPQPDSQTPSAGGADTQTFSSQADNPTLTPAPGGGTDPPGVPGERTVPGTIPADTAFPVDTPSLARNSSAASPTHTSNVSTTDISSGASLTTLTPSTLGLASTDPPSTTIATTTKQTCAAMFGNITVNYTyESSNQTFKADLKDVQNAKCGNEDCENVLNNLEECSQIKNISVSNDSCAPATTIDLYVPPGTDKFSLHDCTPKEKANTSICLEWKTKNLDFRKCNSDNISYVLHCEPENNTKCIRRNTFIPERCQLDNLRAQTNYTCVAEILYRGVKLVKNVINVQTDLGIPETPKPSCGDPAARKTLVSWPEPVSKPESASKPHGYVLCYKNNSEKCKSLPNNVTSFEVESLKPYKYYEVSLLAYVNGKIQRNGTAEKCNFHTKADRLFSQWSVRGRFSYKK* |

^1^ Color coding. Purple: added Restriction sites; Orange: LoxP sites; Red: Exon 14, Bold: position ddPCR

**Supplementary table 2. Antibodies used for flow cytometric analysis.**

| Antigen | Conjugate | Clone | Dilution | Company |
| --- | --- | --- | --- | --- |
| 2B4 | FITC | m2B4(B6)458.1 | 1:50 | BD |
| B220 | Biotin | RA3-6B2 | 1:200 | Biolegend |
| B220 | AF700 | RA3-6B2 | 1:400 | WEHI antibody facility |
| CD105 | PE | MJ718 | 1:200 | eBioscience |
| CD11b | BV605 | M1/70 | 1:400 | BD |
| CD11b | BV605 | M1/70 | 1:400 | BD |
| CD11c | Biotin | HL3 | 1:200 | Biolegend |
| CD122 | BUV661 | TM-BETA-1 | 1:100 | BD |
| CD127 | BUV737 | SB/199 | 1:50 | BD |
| CD127 | APC-Cy7 | A7R34 | 1:75 | Biolegend |
| CD150 | BV421 | TC15-12F12.2 | 1:150 | Biolegend |
| CD16/32 | PerCP-Cy5.5 | 2.4G2 | 1:200 | Biolegend |
| CD19 | APC | 1D3 | 1:200 | Biolegend |
| CD19 | Biotin | 1D3 | 1:200 | Biolegend |
| CD19 | AF700 | 1D3 | 1:400 | WEHI antibody facility |
| CD2 | AF700 | RM-1 | 1:400 | WEHI antibody facility |
| CD27 | PE | LG3A10 | 1:200 | BD |
| CD27 | APC-Cy7 | LG3A10 | 1:100 | BD |
| CD3 | Biotin | 145-2C11 | 1:200 | Biolegend |
| CD34 | A647 | RAM34 | 1:50 | BD |
| CD3e | PerCP-Cy5.5 | 145-2C11 | 1:400 | BD |
| CD4 | Biotin | GK1.5 | 1:200 | Biolegend |
| CD4 | AF700 | GK1.5 | 1:400 | WEHI antibody facility |
| CD41 | PE-Cy7 | MWReg30 | 1:300 | Biolegend |
| CD45 | BV786 | 30-F11 | 1:300 | BD |
| CD48 | FITC | HM48-1 | 1:200 | eBioscience |
| CD49a | BV711 | Ha31/8 | 1:100 | BD |
| CD49b | BV421 | DX5 | 1:100 | Biolegend |
| CD8 | AF700 | 53-6.7 | 1:400 | WEHI antibody facility |
| CD8a | BV650 | 53-6.7 | 1:800 | BD |
| CD8a | Biotin | 53-6.7 | 1:200 | Biolegend |
| CD9 | Biotin | KMC8 | 1:200 | WEHI antibody facility |
| cKit (CD117) | APC | 2B8 | 1:200 | BD |
| cKit (CD117) | BV711 | 2B8 | 1:200 | BD |
| F4/80 | Biotin | BM8 | 1:100 | Biolegend |
| F4/80 | AF700 | BM8 | 1:400 | WEHI antibody facility |
| FLT3 (CD135) | PE | A2F10 | 1:100 | BD |
| FLT3 (CD135) | PE-Cy5 | A2F10 | 1:50 | eBioscience |
| Gr1 | Biotin | RB6‐8C5 | 1:200 | Biolegend |
| Gr1 | AF700 | RB6-8C5 | 1:400 | WEHI antibody facility |
| KLRG1 | PE-Cy7 | 2F1 | 1:400 | eBioscience |
| Ly6G | AF700 | 1A8 | 1:400 | WEHI antibody facility |
| Ly6G (not GR-1) | Biotin | 1A8 | 1:100 | Biolegend |
| MHC-2 | Biotin | M5/114.15.2 | 1:200 | Biolegend |
| NK1.1 | APC-Cy7 | PK136 | 1:100 | BD |
| NK1.1 | BV650 | PK136 | 1:100 | BD |
| NK1.1 | AF700 | PK136 | 1:400 | WEHI antibody facility |
| NKp46 | PE-Cy7 | 29A1.4 | 1:50 | eBioscience |
| NKp46 | BV421 | 29A1.4 | 1:100 | Biolegend |
| Sca1 | A594 | E13-161-7 | 1:400 | WEHI antibody facility |
| Sca1 | A594 | E13-161-7 | 1:400 | WEHI antibody facility |
| Streptavidin | FITC |  | 1:200 | BD |
| Streptavidin | BV510 |  | 1:300 | BD |
| Streptavidin | BV650 |  | 1:300 | BD |
| TCRβ | PerCP-Cy5.5 | H57-597 | 1:400 | BD |
| TER119 | Biotin | TER-119 | 1:200 | Biolegend |
| TER119 | AF700 | TER-119 | 1:400 | WEHI antibody facility |
